# Barriers to accessing follow up care in post-hospitalized trauma patients in Moshi, Tanzania: A mixed methods study

**DOI:** 10.1371/journal.pgph.0000277

**Published:** 2022-06-13

**Authors:** Anjni Patel Joiner, Anna Tupetz, Timothy Antipas Peter, Julius Raymond, Victoria Gerald Macha, João Ricardo Nickenig Vissoci, Catherine Staton

**Affiliations:** 1 Division of Emergency Medicine, Department of Surgery, Duke University School of Medicine, Durham, NC, United States of America; 2 Duke Global Health Institute, Duke University, Durham, NC, United States of America; 3 Kilimanjaro Christian Medical Centre, Moshi, Tanzania; BRAC University, BANGLADESH

## Abstract

Disproportionately high injury rates in Sub-Saharan Africa combined with limited access to care in both the acute injury phase and for injury patients requiring continued care after hospital discharge remains a challenge. We aimed to characterize barriers to transportation and access to care in a cohort of post-hospitalized injury patients in Moshi, Tanzania. This was a mixed-methods study of a prospective cohort of trauma registry patients presenting to Kilimanjaro Christian Medical Center between August 2018 and January 2020. We conducted standardized patient/family surveys and in-depth interviews at a 2-week follow up visit after hospital discharge, and focus groups with healthcare providers. Quantitative results were analyzed using descriptive statistics and multivariable logistic regression using R statistical software. Qualitative results were analyzed using thematic analysis through an iterative process using NVivo software. A total of 1,365 patients were enrolled in the trauma registry, with 169 patients followed up at 2 weeks. Over half of patients at follow-up, 101 (59.8%), reported challenges in traveling. The majority of patients were male (80.3%). Difficulty in traveling since injury was associated with female gender (aOR 5.85 [95% CI 1.20–33.59]) and a need for non-family members escorts for travel (aOR 7.10 [95% CI 1.43–41.66]). Those who reported assault or fall as the mechanism of injury as compared to road traffic injury and had health insurance were less likely to report challenges in traveling (aOR 0.19 [95% CI 0.03–0.90]), 0.11 [95% CI 0.01–0.61], 0.14 [95% 0.02–0.80]). Transportation barriers that emerged from qualitative data included inability to use regular means of transportation, financial challenges, physical barriers, rigid compliance to physician orders, access to healthcare, and social support barriers. Our findings demonstrate several areas to address transportation barriers for post-injury patients in Tanzania. Educational interventions such as clarification of doctors’ orders of strict bedrest, provision of vouchers to support financial challenges and alternate means of transportation given physical barriers and reliance on social support may address some of these barriers.

## Introduction

It is estimated that 10% deaths and 16% of all disabilities worldwide are caused by injury [1]. Low and middle-income countries (LMIC) share the burden of over 90% of unintentional injuries and over 90% of disability life-adjusted years in the world [2]. These injuries can lead to temporary or long-term disabilities in those affected [3–6]. One billion of the world’s population are considered disabled, and between 110–190 million people encounter difficulties in their daily lives [4,7]. Sub-Saharan Africa is a region with some of the highest rates of injuries in the world and limited prehospital and rehabilitation care [4,8]. For these patients, access to care both at the acute injury phase and for the injured patient requiring continued care after hospital discharge, remains challenging.

Given that injured patients are 20 to 50 times more likely to develop disabilities compared to death, continued follow-up and rehabilitation after injury is crucial to preventing further complications and expediting recovery [9,10]. Low- and middle-income countries have limited or non-existent rehabilitative services or follow-up care compared to high-income settings [4]. Moreover, in areas where these services exist, access for those with disabilities can be challenging, with cost, physical barriers, and mode of transport cited as common limiting factors [4,11,12]. However, we know that barriers to accessing outpatient care for other conditions in LMIC represent a complex interplay of multiple factors [13].

In recently hospitalized injury patients, an in-depth understanding of barriers to accessing follow-up care is key in developing effective strategies for increasing follow-up compliance, adherence to rehabilitative services, and subsequent recovery. This study aims to identify barriers in accessing follow-up care in the post-hospitalized injury population in Moshi, Tanzania.

## Methods

### Ethics statement

This study protocol was approved by the Duke University Medical Center Institutional Review Board under the IRB protocol number Pro00086496 and the Kilimanjaro Christian Medical Center Ethics Committee and the Tanzanian National Institute of Medical Research (NIMR). Written informed consent was obtained in Swahili from all participants involved in the study, including both qualitative and quantitative (2-week follow ups). Data from the trauma registry did not require informed consent as it was collected as part of an ongoing quality improvement process approved by KCMC and NIMR.

**Fig 1:**
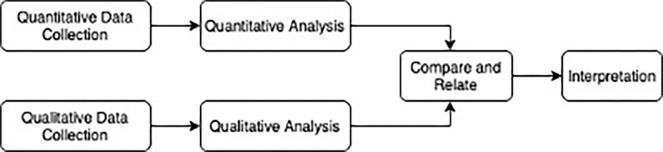
Triangulation of qualitative and quantitative data from collection to interpretation.

We used a mixed methods convergent parallel design to gain an in-depth understanding of transportation challenges for injury patients after hospitalization. The quantitative portion consisted of a secondary analysis of a prospective cohort from trauma registry survey data collected at the time of hospitalization and two weeks after discharge from the hospital. The qualitative portion consisted of focus group discussions with patients, family members, and healthcare providers. Both data sets were obtained and analyzed independently, and the results were triangulated for direct comparison (Fig 1).

### Setting

Moshi Municipal Council and Moshi Urban Council are two districts within the Kilimanjaro region in Northern Tanzania. The remaining districts in the region are Hai, Siha, Same and Mwanga district councils with total populations of 1,640,087 from 2012 census data [14]. The area contains a mixture of paved versus mud roads, and often vehicles with 4-wheel drive are required to cross the rugged terrain in more rural areas. The most common forms of public transportation are dala dalas (mini buses) and other alternate forms of transport include boda bodas (motorcycle taxis), bijajis (3-wheeled taxis), and taxi cars. Typical costs for using public transportation for a cross-town trip are less than 5,000 Tanzanian shillings (approximately 2 US dollars), with taxi cars being the most expensive.

The study was conducted at Kilimanjaro Christian Medical Centre (KCMC), an urban tertiary referral hospital located in Moshi Municipal council. With a hospital capacity of more than 640 beds, the catchment area for KCMC spans four regions: Kilimanjaro, Arusha, Tanga, and Manyara. The hospital system provides both inpatient and outpatient care in multiple specialties. The KCMC Emergency Department is staffed by trained emergency medicine physicians and sees approximately 2,000 injury patients annually [15].

### Quantitative

#### Data collection and study instruments

Data were collected at initial hospitalization as a part of a prospective trauma registry (S1 Data) and again at a 2-week follow-up via administration of a standardized survey tool that was developed as a part of a larger study on patient care transitions into the community after injury (S2 Data). Data were collected between August 2018 and February 2020. Participants were screened for participation at the time of entry into the trauma registry.

The 2-week follow-up survey was developed with input from two physicians from the United States, a physician from Tanzania, a physical therapist and the local community advisory board (CAB). The tool was then reviewed for content validity, format, and cultural appropriateness by the local Tanzanian emergency medicine research team of trained research nurses and research assistants. The survey was piloted by the local research team prior to implementation in order to ensure correct language translation, cultural appropriateness, and to be fully inclusive of all local means of transport.

The survey tool was initially developed in English and then translated into Swahili by bilingual research team members. The tool was administered in Swahili by trained research assistants at the patient’s home, hospital clinics, or via phone interview.

#### Study participants

Patients enrolled in the prospective trauma registry were screened for a two-week post-discharge follow-up survey. Inclusion criteria for the initial trauma registry were: 1) age ≥ 18 years, 2) had an injury requiring hospitalization within 24 hours of presentation, 3) initial visit for the injury. Patients enrolled in the trauma registry were then further screened for participation in the two-week follow-up study if they resided in Moshi Urban, Moshi Rural, or nearby (defined as an estimated cost of 5,000 Tanzanian shillings for transportation from their home to KCMC). Patients who were discharged to a location other than their usual home environment, such as prison or a substance abuse rehabilitation center, were excluded.

#### Variables

Demographic information and variables specific to the injury and prehospital course were collected from the prospective trauma registry and included the following: age, sex, district of residence, insurance, employment status, mechanism of injury, mechanism of arrival, initial health centre to which the patient presented and injury intent. Data on hospital course included: ICU stay, length of stay in the ICU and hospital, number of surgical interventions, Glasgow Coma Score (GOS), and a modified Functional Independence Measure (FIM) (S1 Data), designed to measure disability. The modified FIM was completed by either the patient or a family member with results categorized as “dependent” (score in the lowest quartile, <51) or “independent” (any score greater than 51).

The two-week follow-up survey collected data from both open and closed-ended questions on the patients’ ability to travel alone, means of transport to appointments, the need for an escort to travel, social support and functional independence. The primary outcome, difficulty in traveling since the injury, was defined as barriers the patients encountered in traveling after hospitalization and was measured through the patient’s self-reported answer (Yes or No) to the following question: “Is travel more difficult since your injury?”. Additional questions were asked to further specify if the challenges were due to financial, logistical, or other reasons. Social support was measured through the modified Medical Outcomes Study Social Support survey (mMOS-SS) (S2 Data), an eight-question survey designed to determine emotional and tangible social support in patients with chronic illnesses. Results from the mMOS-SS were dichotomized, with scores in the lower quartile (less than 21) considered “poor support” and any scores above this categorized as “good support”.

#### Data analysis

Statistical analysis was performed using R statistical software, Version 4.0.2. Categorical variables were expressed as frequencies and percentages, continuous variables were expressed as mean and standard deviation (SD). The associations between the outcome of interest, difficulty in traveling after injury, and the predictors described earlier were assessed using logistic regression. Univariable and multivariable models were developed in an exploratory analysis approach. The association between the selected predictors and outcomes are reported in odds ratios and 95% confidence intervals. No elimination methods were applied and models were built by building predictors that were deemed to theoretically be associated with difficulty in traveling since the injury. Missing data were analyzed and deemed to be missing at random, thus a complete case analysis was performed in the multivariable models.

### Qualitative analysis

#### Study design and theoretical framework

We used a phenomenological approach to focus on the individual experiences of the patients, caregivers, and healthcare personnel. The study adopted a longitudinal qualitative research using a multi-perspective approach in order to acquire dynamic experience on barriers for transportation for injury patients post hospitalization.

#### Data collection

Qualitative data via focus groups and in-depth interviews were collected between January 20, 2019 and December 12, 2019. Five Tanzanian research assistants, three females and two males with either a bachelor’s or master’s degree and a registered nurse all with training in qualitative data collection, collected the data through in-depth interviews and focus group discussions. A sample size of 45 patients and caregiver interviews was estimated. If thematic saturation of qualitative data was reached prior, data collection was terminated.

Interviews lasted approximately 30 minutes on average, but ranged between 10 and 70 minutes. Interviews were conducted in Swahili in a private room at KCMC or at the patient’s home; only the participant and interviewer was present when the interview was taking place. The researchers informed the participants on the study process and goals at the beginning of data collection. The researchers also emphasized that they were not involved in the clinical care of the patient, nor would their participation in the study affect the patient’s treatment or hospital care. Interviews were audio-recorded on encrypted devices and uploaded onto a secure database behind a firewall on a university database. Audio recordings were deleted from the devices after being uploaded.

#### Study instruments

Semi-structured interview guides for both the in-depth interviews and focus groups (S1 and S2 Text) were developed iteratively with input from mixed methods researchers from both institutions. The interview guides were initially created in English and then translated and back-translated by bilingual Tanzanian research assistants. The local Tanzanian research team evaluated the interview guides for cultural appropriateness and piloted the instruments prior to implementation. The interview guides were reviewed by the research team mid-way through data collection and revised based on preliminary results.

#### Study participants

Convenience sampling was used for in-depth interviews with patients as they were identified at the time of entry into the trauma registry. Additional data were collected on the barriers to transportation through interviewing the patients’ family members and two focus group discussions with health care providers, and Community Advisory board (CAB) members. Inclusion criteria for patients included the following: 1) age ≥ 18 years, 2) medically stable, 3) living in or near Moshi Urban or Moshi Rural and 4) injuries requiring hospitalization. Patients who were not discharged to their usual home environment, such as patients who were incarcerated or admitted to a substance use rehabilitation center, were excluded. Patient caregiver inclusion criteria were, 1) age ≥ 18 years, 2) residing close to the patient after discharge. Any CAB member 18 years or older was eligible for inclusion.

#### Data analysis

Interviews were transcribed ad verbatim in Swahili to ensure consistency and then translated to English by bilingual Tanzanian research assistants. The initial codebook was developed by a qualitative researcher following a mix of deductive and inductive analysis approaches using the first round of interviews. The coding was done by three data analysts, and the emergent codebook was developed and updated throughout the data collection process through discussions between data analysts and the interviewers, to validate the interpretation of our findings and to validate the developed themes.

The de-identified transcripts were coded and analyzed using google spreadsheets, which allowed for close collaboration and communication between the research team in different locations by providing comments and posing questions throughout the coding process.

## Results

### Quantitative

**Fig 2:**
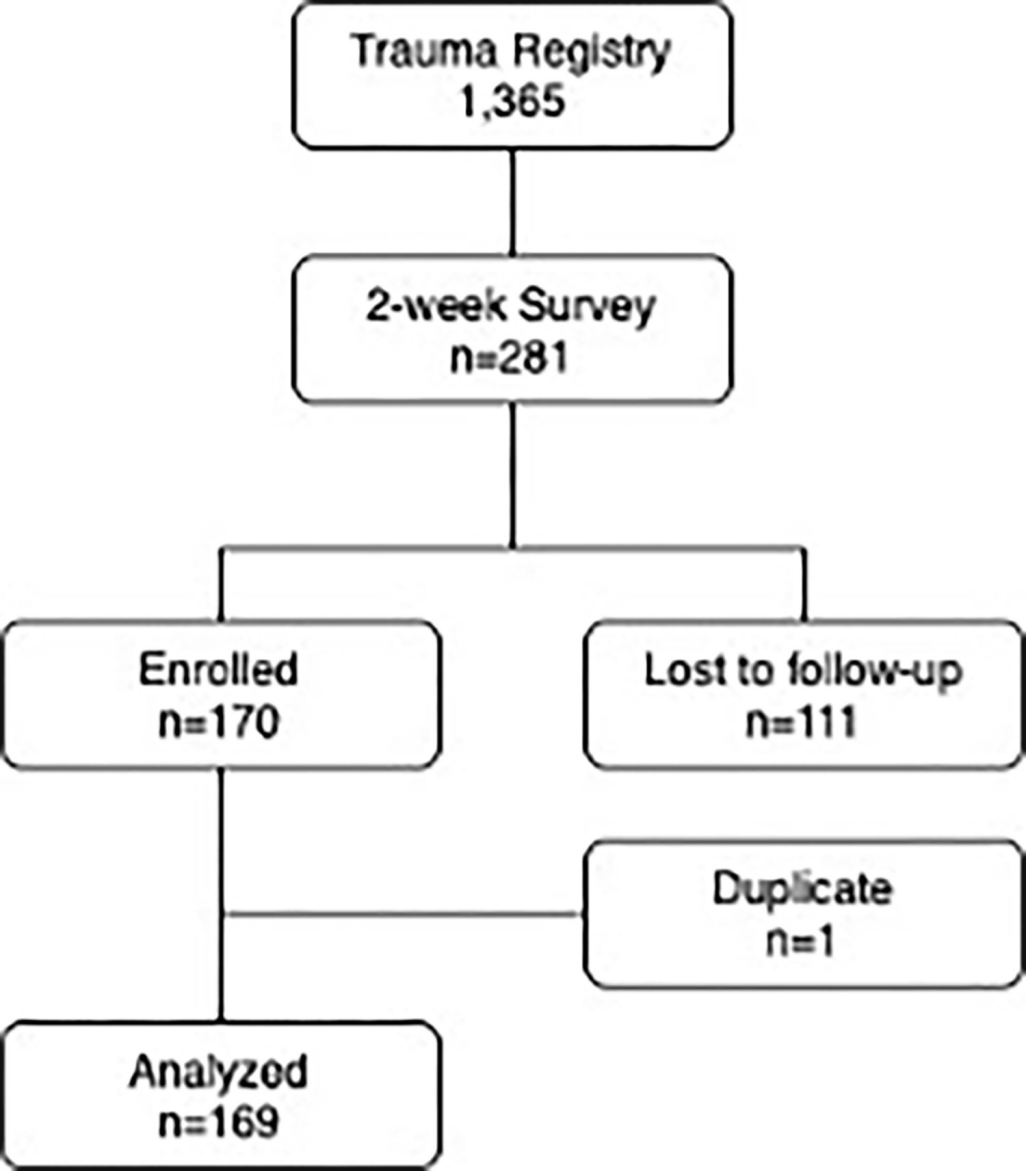
Enrollment flow diagram.

A total of 1,365 patients visited the KCMC emergency department over the course of the study due to injuries (Table 1). Of those patients, 281 met inclusion criteria and consented to participate in the study (Fig 2). Of the 281, 169 were followed up at two weeks, of which more than half reported experiencing difficulties in traveling after their injury 101 (59.8%) (Table 1). The average age of those followed up at 2 weeks was 39.8 years. The large majority of patients were males (80.3%). Males and females had similar proportions of reported difficulty in traveling since their injuries, 61.8% and 59.7%, respectively. The majority of patients (44.0%) resided in Moshi rural, followed by 18.5% in Moshi urban and 37.5% from other districts. Most patients were uninsured (82.0%) and self-employed (65.5%). Road traffic injury comprised 64.5% of all injuries with 65.1% of those patients reporting having difficulty traveling after the injury. Only 10.8% of injury patients were treated in the intensive care unit (ICU), however, approximately two-thirds of those who did go to the ICU reported difficulty in traveling as compared to 59.5% of those who did not require ICU care. Of those who underwent surgery, 61.8% reported difficulty in travel after injury compared to 55.9% of those who did not undergo surgery. Most patients who had surgery (93.6%) underwent only one surgery. Of these patients, 60.2% reported difficulty traveling whereas 85.7% of those who underwent more than one surgery reported difficulty traveling. The median Glasgow Outcome Scale Extended (GOS-E) for each category was 7, which corresponds to “lower good recovery”, indicating minor physical or mental deficits. The functional independence measure (FIM) at discharge was similar for both groups as well, with a median score of 60 for those who reported difficulty in traveling, and 62 for those who reported no difficulty in traveling after their injury.

**Table 1:** Demographics, injury characteristics, clinical/functional variables and transportation/support information for trauma patients during hospital stay and at two week follow-up.

		Two week follow up cohort (n = 169)	
**Variable**	Baseline (Trauma Registry) N = 1365*	**Difficulty in traveling since injury (N = 101)**	**No difficulty in traveling since injury (N = 68)**
**Demographics**			
Age, mean (sd)	38.48 (16.41)	39.51 (18.37)	39.24 (15.82)
**Gender n (%)**			
Male	1106 (81.0)	80 (59.7)	54 (40.3)
Female	251 (18.4)	21 (61.8)	13 (38.2)
Missing (n)	8 (0.6)	0	1
**Residence, n (%)**			
Moshi urban	317 (23.2)	18 (58.1)	13 (41.9)
Moshi rural	515 (37.7)	44 (59.5)	30 (40.5)
Other	525 (38.5)	38 (60.3)	25 (39.7)
Missing	8 (0.6)	1	0
**Type of insurance, n (%)**			
No Insurance	1150 (84.2)	84 (61.3)	53 (38.7)
With Insurance	202 (14.8)	16 (53.3)	14 (46.7)
Missing	13 (1.0)	1	1
Employment status n (%)			
Unemployed	94 (6.9)	8 (66.7)	4(33.3)
Employed	328 (24.0)	24 (60.0)	16 (40.0)
Self Employed	871 (63.8)	66 (60.0)	44 (40.0)
Others	50 (3.7)	3 (50.0)	3 (50.0)
Missing	22 (1.6)	0	1
**Injury Characteristics**			
**Mechanism of Injury, n (%)**			
Road Traffic Injury	942 (69.0)	71 (66.0)	38 (34.0)
Assault	189 (13.8)	10 (41.7)	14 (58.3)
Fall	227 (16.6)	20 (55.6)	16 (44.4)
Missing	7 (0.5)	0	0
**Mechanism of Arrival n (%)**			
Ambulance from other Hospital	892 (65.3)	62 (62.0)	38 (38.0)
Police car	86 (6.3)	7 (41.2)	10 (58.8)
Hired Transportation	325 (23.8)	26 (60.5)	17 (39.5)
Personal transport	31 (2.3)	3 (50.0)	3 (50.0)
Others	13 (1.0)	2 (100.0)	0 (0.0)
Missing	18 (1.3)	1	0
**Treated at KCMC first n (%)**			
Yes	306 (22.4)	28 (59.6)	19 (40.4)
No	1046 (76.6)	73 (60.3)	48 (39.7)
Missing	13 (1.0)	0	1
**Intention of Injury n (%)**			
Intentional	156 (11.4)	8 (44.4)	10 (55.6)
Non-Intentional	1179 (86.4)	91 (61.5)	57 (38.5)
Unknown	11 (0.8)	1 (50.0)	1 (50.0)
Missing	19 (1.4)	1	0
**In-hospital care**			
**Variable**	**Baseline (Trauma Registry) N = 1365**	**Difficulty in traveling since injury (N = 101)**	**No difficulty in traveling since injury (N = 68)**
**ICU n (%)**			
Yes	124 (9.1)	12 (66.7)	6 (33.3)
No	1193 (87.4)	88 (59.5)	60 (40.5)
Missing	48 (3.5)	1	2
**Length of stay in the Hospital (Days) n (%)**			
< 6 days	535 (39.2)	39 (60.0)	26 (40.0)
≥ 6 days	688 (50.4)	49 (57.0)	37 (43.0)
Missing	142 (10.4)	13	5
**ICU stay**	**Baseline (Trauma Registry) n = 124**	**Difficulty in traveling since injury (n = 12)**	**No difficulty in traveling since injury (n = 6)**
**Length of stay in the ICU(Days) n (%)**			
< 6 days	56 (45.2)	4 (50.0)	4 (50.0)
≥ 6 days	21 (16.9)	3 (75.0)	1 (25.0)
Missing	47 (37.9)	5	1
**Had a surgery n (%)**			
Yes	866 (63.4)	68 (61.8)	42 (38.2)
No	464 (34.0)	33 (55.9)	26 (44.1)
Missing	35 (2.6)	0	0
**Surgery**			
**Surgeries n (%)**	**Baseline (Trauma Registry) n = 866**	**Difficulty in traveling since injury (n = 68)**	**No difficulty in traveling since injury (n = 42)**
1	781 (90.2)	62 (60.2)	41 (39.8)
>1	85 (9.8)	6 (85.7)	1 (14.3)
Missing	0	0 (0.0)	0 (0.0)
**GOS at Discharge Median (IQR)**	7 (7–8)	7 (7–8)	7 (7–8)
**FIM at discharge Median (IQR)**	61 (52–68)	62.00 (51.25–69.00)	60 (50.25–70.00)
**Dependent (<51 of total scores)**		23 (56.1)	18 (43.9)
**Independent (> = 51 of total scores)**		67 (58.3)	48 (41.7)
**Transportation and support at two weeks**			
**Social support (mMOS-SS)**			
Poor support (<26 of total scores)	-	24 (53.5)	21 (46.7)
Good support (> = 26 of total scores)	-	76 (63.9)	43 (36.1)
**Escort to travel**			
No one	-	10 (28.6)	25 (71.4)
Family members	-	22 (55.0)	18 (45.0)
Others	-	18 (69.2)	8 (30.8)
**Ability to travel alone**			
No	-	67 (73.6)	24 (26.4)
Yes	-	34 (44.2)	43 (55.8)
**Means of transport to the appointment**			
Cars	-	30 (49.2)	31 (50.8)
Motorcycle	-	19 (50)	19 (50)

In the multivariate adjusted model (Table 2) we have found that difficulty traveling since injury was associated with gender, mechanism of injury and people who required an escort to travel to their follow-up appointment. People with health insurance had a lower odds of reporting difficulty in transportation compared to those without health insurance. Females had a 5.85 times increased adjusted odds of reporting difficulties in traveling after injury compared to males (95% CI 1.20–33.59). Patients with assault or falls as their mechanism of injury reported a lower odds of having difficulties in traveling since their injury when compared to falls or assaults. Patients who required an “other” escort to their follow up appointments were found to have a greater than 7 times higher adjusted odds of reporting difficulty in traveling since injury compared to patients who traveled alone or with family members (95% CI 1.43–41.66). Patients with the ability to travel alone were also less likely to report having difficulties in traveling since injury.

**Table 2:** Multiple regression model of patient demographics associated with difficulties in transportation.

	Difficulty traveling since injury, n (%)	No difficulty traveling since injury, n (%)	OR (95% CI)	AOR (95% CI)
**Age (Years)**				
18–35	53 (58.2)	38 (41.8)	REF	REF
36–50	32 (58.2)	23 (41.8)	1 (0.51–1.98)	1.25 (0.29–5.66)
> 60	16 (69.6)	7 (30.4)	1.64 (0.63–4.62)	1.71 (0.23–14.21)
**Gender**				
Male	80 (59.7)	54 (40.3)	REF	REF
Female	21 (61.8)	13 (38.2)	1.09 (0.51–2.41)	5.85(1.20–33.59)
**Residence**				
Moshi Urban	18 (58.1)	13 (41.9)	REF	REF
Moshi Rural	44 (59.5)	30 (40.5)	1.05 (0.44–2.44)	0.46 (0.09–2.09)
Other	38 (60.3)	25 (39.7)	1.10 (0.45–2.63)	1.16 (0.24–5.70)
**Insurance**				
No health insurance	84 (61.3)	53 (38.7)	REF	REF
Health insurance	16 (53.3)	14 (46.7)	0.72 (0.32–1.61)	0.14 (0.02–0.80)
**Mechanism of injury**				
Road traffic injury	71 (66.0)	38 (34.0)	REF	REF
Assault	10 (41.7)	14 (58.3)	0.38 (0.15–0.93)	0.19(0.03–0.90)
Fall	20 (55.6)	16 (44.4)	0.67 (0.31–1.45)	0.11 (0.01–0.61)
**Employment Status**				
Employed	24 (60.0)	16 (40.0)	REF	REF
Self-employed	66 (60.0)	44 (40.0)	1.00 (0.47–2.08)	0.61 (0.12–2.84)
Unemployed	8 (66.7)	4 (33.3)	1.33 (0.36–5.68)	2.54 (0.20–39.33)
Other	3 (50.0)	3 (50.0)	0.67 (0.11–4.00)	1.99(0.11–40.11)
**Ability to travel alone**				
No	67 (73.6)	24 (26.4)	REF	REF
Yes	34 (44.2)	43 (55.8)	0.28 (0.15–0.54)	0.33(0.08–1.16)
**Means of transport to the appointments**				
Cars	30 (49.2)	31 (50.8)	REF	REF
Motorcycle	19 (50)	19 (50)	1.03 (0.46–2.33)	1.63 (0.52–5.50)
**Escort to travel**				
No one	10 (28.6)	25 (71.4)	REF	REF
Family members	22 (55.0)	18 (45.0)	3.06 (1.19–8.25)	3.33 (0.69–18.20)
Others	18 (69.2)	8 (30.8)	5.62 (1.92–17.94)	7.10 (1.43–41.66)
**Social Support**				
Poor Support	24 (53.3)	21 (46.7)	REF	REF
Good Support	76 (63.9)	43 (36.1)	1.55 (0.77–3.10)	1.52 (0.36–6.47)
**FIM at discharge**				
Dependent	23 (56.1)	18 (43.9)	REF	REF
Independent	67 (58.3)	48 (41.7)	1.09 (0.53–2.24)	2.94(0.79–12.79)

### Qualitative

A total of 26 participants in the patient group (male, n = 21) and 11 participants in the caregiver group (male, n = 4) were interviewed. Focus group discussions had a total of 46 participants in total: 37 participants from the monthly Community Advisory Board meeting and 9 participants from the separate healthcare provider focus group, which included 7 nurses, 1 medical doctor, and 1 physiotherapist.

The most common transportation barriers that emerged from this study from patients and family members were the inability to use regular means of transportation and financial challenges associated with the transportation process. Some patients also identified physical barriers resulting from their injuries as a challenge in using transportation. Healthcare providers indicated that access to healthcare was affected by transportation barriers (See Table 3). Other barriers identified included strict compliance to doctor’s orders for bedrest resulting in limited movement and transportation, challenges with assistive devices, and inability to travel alone. Patients also identified personal factors as barriers to transportation, such as a fear of reinjury and family problems.

**Table 3:** Qualitative barriers to transportation after injury.

Themes	Categories	Codes
Financial challenges and transportation costs	Need to use private transportation means	Hiring a driver Hiring a taxi Hiring bajaji Finding money for private transportation
	Treatment costs hinders transportation	Finding money for hospital care
Physical ability		Cannot walk for a long time Cannot bend their leg Leg swells on long routes Dizziness Not feeling well Pain Cannot sit for a long time
Ability to use particular means of transportation		Finding a private car (inability to use public transportation) Use transportation that moves slowly Use modified means of transportation Need transportation with space
Personal factors		Fear of reinjury Family problems
Compliance to doctor’s orders	Movement limited as adherence to doctor’s order	Doctor ordered full time bed rest Doctor said not to move the leg so much
Personal/educational/occupational commitment		Need to attend school
Assistive devices	Needing assistive device	Needs pillow to avoid pain
	Difficulties in using assistive devices	Difficulties in using crutches
Social challenges	Community support	Needs someone to travel with them to the hospital Needs someone to hold their leg
	Family support	need someone to hold their hands, take them to hospital
Access to healthcare	Lack of nearby health facilities	Lack of nearby health facilities (perceived level of quality of medical care received)
	Knowledge of healthcare access and resources	Lack of knowledge of nearby facilities to reduce travel requirements
	Limited healthcare provider availability	Limited physicians at clinics

#### Financial challenges and transportation costs

Challenges in obtaining transportation due to financial constraints were reported by participants in both the interviews and focus groups as barriers to transportation in recently injured patients. Patients, family members and health care providers mentioned financial challenges and transportation costs as limiting factors in continuing treatment even with the knowledge that it was necessary.

Participants in both interviews and surveys reported that most injury patients had to find extra money for hiring means of transportation resulting in increased financial burden to the patients. They indicated that an inability to use public transportation forced the patient to hire bajajis and taxis for transportation to follow up clinics. Also, the fact that the patient was not able to work, resulting in a loss of income made it even harder to afford the cost of transportation. These challenges are reflected in the following quote:

"*[…] not much because the way we bring him here we must use a means, and that transportation is taxi, and that is the only thing that can carry him otherwise nothing carry him and the taxi has to be paid for*, *it is not free and that also is a challenge" (Family IDI 111)*

#### Physical abilities

Participants in interviews and surveys identified physical limitations as a barrier to transportation. Examples included patients not being able to bend their leg after injury or leg swelling when travelling on long routes. In one interview, a patient stated that he had to send someone to bring a bajaji to him as he was physically unable to hail one. The following quotes demonstrate examples of these types of barriers:

“*I can’t go very far because if I walk for long my arm aches*" *(Patient IDI 567)*

“*Yeah, because I have neighbors, the day that my wife hasn’t gone out*, *I just send someone, the bajaji pass here often, the person goes to stand there and wave and come with it, even our neighbors here have three bajaji” (Patient IDI 593)*

#### Ability to use regular mode of transportation

Most of the participants in interviews, surveys and focus groups identified an inability to use their regular mode of transportation as a barrier to transportation for injury patients. A patient in an interview said he used to rely on his motorcycle for transportation and now he has to hire a bajaji. Several patients had to change from their usual means of transportation to more comfortable means. The following quote represents this challenge.

“*Transportation is the problem because I was relying on my motorcycle and that’s not working anymore*, *here I just hire a Bajaji when I am going for wound dressing at Msaranga Dispensary.” (Patient IDI 423)*

“*Transportation, we had to be using the public transport, it also affects income because you find sometimes he used to go alone and come back but now you have to pay someone to go with him or sending someone to look for needs in the market*.” *(Family 240)*

#### Access to health care

Health care providers and CAB members mentioned that access to health care was affected by transportation challenges. One CAB member believed that transportation to receive medical care was the first challenge in accessing healthcare, since medical services are not close enough to be accessed without modes of transportation. Additionally, one patient mentioned that transportation was required in order to access specialized care. This was reiterated by a healthcare provider focus group participant who stated that a lack of healthcare specialists, particularly for those with chronic or long-term rehabilitation or medical needs, the cost of public transportation and the time commitment required in order to travel long distances to access specialized care were all barriers to access. Another healthcare provider mentioned the need to educate patients on nearby health facilities to limit the need for transportation and thereby improve access to care and follow up appointments. Overall, healthcare providers identified a lack of proximity to healthcare and the need for specialized services as barriers that ultimately limit access to care.

“…*the relevant institutions should ensure that we have the enough staff to serve those patients who come to us instead of getting access to other low level centres*, *for example the expansion and understanding of the service available to the patient who has suffered injuries” (healthcare provider)*

#### Compliance to doctors’ orders

Healthcare providers stated that when patients live too far to properly follow up with them, there is an increased risk of patients not adhering to medication schedules, resulting in complications. Lack of transportation was also mentioned as a limiting factor to accessing medications by both patients and caregivers.

Patients in surveys also mentioned that they were instructed not to move or travel and had to comply with their doctor’s order for full time bed rest, thus limiting their ability to travel.

“*Doctor restrictions (full time bed rest)*” *(patient survey)*

“*I was told not to move this leg so much*” *(patient survey)*

“[…] *a doctor must require you when you sit you have to put your legs straight, even when I will be in the car legs must be kept straight*, *I use the elongated back seat as precaution […] I can’t sit like other normal passengers.” (P577)*

#### Assistive devices

Patients in interviews described the need to use assistive devices for movement, which in some cases served as barriers in using certain modes of transportation. Patients in surveys stated that they needed to use assistive devices like crutches or wheelchairs for moving around and sometimes had difficulties using them.

“*I have to sit on a wheelchair and get into the car with a lot of pillows*” *(Patient survey)*

“*Using the crutches was hard at first*, *getting tired and falling” (Patient survey)*

#### Social support

Participants in most of the interviews and surveys mentioned social support as an important factor in transportation for injury patients. Dependence on family support and community as a whole was brought up by both patients and relatives in interviews and surveys when transportation was being discussed.

Participants in interviews and surveys mentioned that transportation required support from both the community and family. Some patients indicated that they were unable to travel to the clinic without the help of family members or friends. These family members or friends would assist the patients in ambulating by holding their hands when they experienced pain or dizziness. Some required assistance in holding their legs during transportation to avoid excessive movement on rough roads. The following quotes highlight this need:

“*Asking for transportation help from friends*” *(Patient survey)*

“…*it is the bajaji, it comes to pick me up and my wife takes me there direct*, *we get to the gate and she takes my hand and we go to the eye ward” (Patient IDI 593)*

#### Personal factors

Several patients in surveys mentioned that they had difficulties travelling due to personal concerns. Two patients identified a fear of reinjuring themselves as a barrier to transportation.

“*I have to get private transportation because the public transports are usually so congested and so it is easier to re-injure my leg*.” *(Patient Survey)*

“*I don’t travel because I have to be careful not to reinjure myself*” *(Patient survey)*

## Discussion

This study revealed those requiring a non-family member escort to travel had a greater than two times odds of reporting difficulties in traveling as compared to those who could travel alone. This finding was constant throughout the methodologies. Our qualitative results depicted the importance of community member involvement in assisting injured persons primarily to provide tangible support, such as assisting with physical tasks. Our results suggest that individuals with physical (short or long-term) disabilities often require assistance when using any form of transportation in boarding, riding and exiting the vehicle [16]. This distinction between tangible support in low-resource settings is important to note given that family and community support may serve as proxies to assistive devices and alternate transportation means for injured or disabled patients that are less readily available in low-resource settings [17]. Difficulties in traveling for those who could not travel alone may also be due to the type of injury. Patients with lower extremity injuries may have limited mobility and more difficulty travelling alone as compared to those that have minor injuries [4].

Financial challenges were frequently discussed during the in-depth interviews as participants were often unable to use their typical means of transportation and were forced to pay extra for more accommodating means of conveyance, such as taxis. This is also reflected in the quantitative results in which insured patients were less likely to experience challenges in transportation after hospitalization when compared to uninsured patients. Other studies have described economic barriers to outpatient visits in LMIC. An evaluation of transportation barriers to healthcare facilities for surgical conditions in Malawi found that nearly half of all patients surveyed indicated financial barriers to seeking surgical care [18]. Financial constraints are also cited as a barrier to seeking obstetric care in sub-Saharan Africa [19]. Moreover, people with disabilities have cited cost as the primary barrier to receiving health care in LMICs [4,20]. The economic burden of injuries to the patient is not well-documented and does not often account for loss of productivity or subsequent healthcare costs after the initial injury [21]. These types of cost assessments in LMICs are even more scarce. In a Northern Indian cohort, the economic burden of medical care due to injuries was found to extend to at least a year beyond the time of admission, with catastrophic expenditure associated with lower income, inpatient stays greater than 1 week, major surgery, and occupation as a wage laborer [22]. Unexpected costs of transportation in addition to the costs of medical care may further impact the ability of injury patients to receive important follow-up care after hospitalization.

Gender was found to be an important factor associated with difficulty in traveling with injury patients after hospitalization. Females had higher odds of reporting difficulty traveling as compared to men. This finding is consistent with other literature suggesting that women are more likely than men to report difficulties in mobility and findings that women tend to have a lower health-related quality of life after even minor road traffic accidents [23–26]. This disparity in reported challenges with mobility between men and women may also have been due to cultural factors such as men not perceiving their condition as severe enough to seek care or the need to reject any portrayal of weakness in order to adhere to cultural and societal expectations of masculinity [4,27]. These types of behaviors have been explored in other high-income settings with more chronic health conditions, but not in the trauma population in low-income settings and may represent a key area of intervention in health promotion.

During focus groups and surveys, study participants from all categories commented on limitations in accessing specialized care in the region. This reflects previous studies in the region and across Africa. Premkumar et al. estimated that only between 3.05 and 10.62% of people living in Northern Tanzania have access to timely, safe, and affordable orthopedic surgical care, with only 39% having timely access to care (within 2 hours) and only 15–20% of individuals in this region would be able to afford orthopedic surgical care [28]. Only 25% of the population in sub-Saharan Africa is estimated to have access to neurosurgical care within 2 hours [29]. Prior studies have noted a limited number of specialty trained physicians, poor transportation access, and underdeveloped healthcare infrastructure as the primary causes of this gap in access for more emergent specialized care [29–31]. The present study demonstrates parallel findings in patients who have continued needs after their initial injury. As these individuals seek recovery, they again face similar challenges as with their initial emergency. The qualitative analysis also identified some healthcare provider-specific barriers that contributed to reduced post-hospitalization follow-up, such as the need to educate patients on follow-up for nearby health facilities to reduce cost-prohibitive lengthy transports and possible misinterpretation of doctors’ orders resulting in strict bed rest. Similar patterns of gaps in discharge planning and instructions for trauma patients are seen in other settings and may be considered as a focus area for low-cost intervention [32,33].

**Fig 3:**
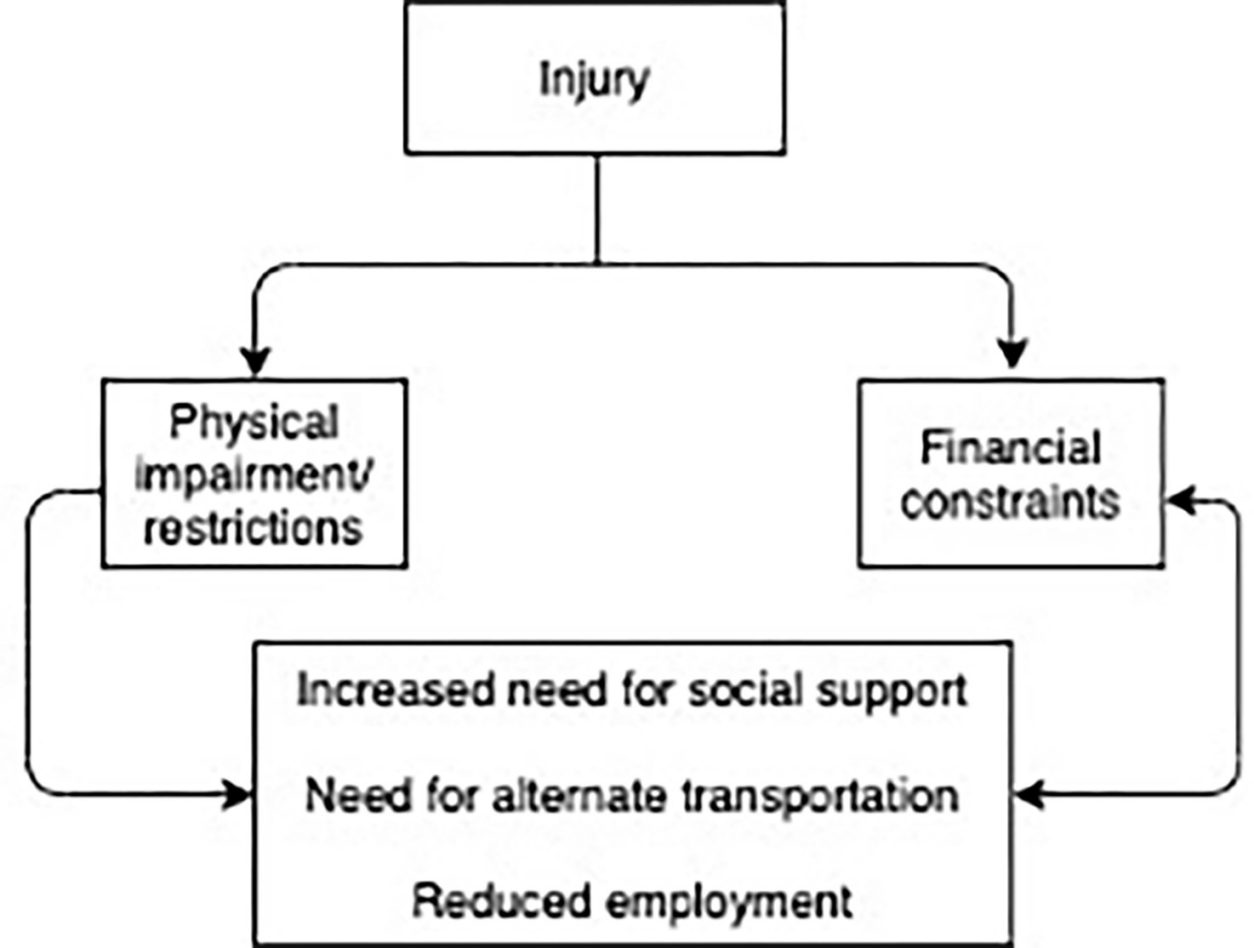
Diagram of transportation barriers in the post-hospitalized injury patient.

Transportation challenges encountered by patients transitioning to home after hospitalization overall appear to be multifactorial and interrelated (Fig 3) [34]. Those who indicate financial barriers may be further challenged with lack of employment due to disability resulting from their injury. Thus perpetuates a cycle of inability to obtain follow-up secondary to high costs of transportation, leading to prolonged or ineffective recovery and subsequent continued lack of employment. This pattern has been noted in road traffic injury patients in other settings as well [35]. Similarly, we found that those injured in road traffic crashes were more likely to report difficulties in traveling compared to other mechanisms of injury. This finding was reinforced in our surveys and in-depth interviews, where we found many patients were unable to use their usual means of transport due to damages to their vehicles from road traffic crashes as well as the injuries to the victims. This represents a significant subset of the injury population that may have additional financial losses or economic implications due to their injury, which could result from loss of their primary means of income if they used their vehicle to earn money or an inability to continue to use their pre-injury employment-related mode of conveyance due to physical limitations from the injury.

Addressing these barriers in low-income settings may require significant infrastructure changes to the larger healthcare system. Given limited resource settings like Moshi, Tanzania, solutions employed in high-income settings, such as dedicated non-emergent medical transport vehicles and readily available assistive devices, such as wheelchairs, may not be applicable. Therefore, focusing on cost-effective solutions such as creating complex coordinated care plans upon hospital discharge and providing detailed patient and caregiver discharge education may be of immediate benefit [36]. Interventions to decrease the financial burden placed on patients after injury, such as investing in unconditional vouchers or reduced cost transportation schemes with vehicles equipped to accommodate a physically disabled patient population also warrant further investigation.

This study had several limitations. Firstly, we encountered a follow-up rate of 60% in this patient population, which limited our overall sample size. Ability to reach patients in this population can prove challenging. Although the trauma registry encompassed all patients presenting to KCMC, our study population was limited to those living in or close to Moshi, Tanzania. Therefore, injury patients residing in more rural areas were excluded. Given even more limited resources to transportation and healthcare facilities in rural areas, this patient population may have experienced different challenges compared to our more urban cohort. Our patient population and data collection from a single tertiary care hospital limits the generalizability of the results, particularly for regions with fewer resources. Our study is also limited to the immediate post-hospitalization period, which fails to encompass some of the prolonged transportation challenges encountered by this population.

## Conclusion

Females and patients with road traffic injuries were more likely to report challenges in transportation after their injuries. Barriers to transportation challenges in the post-hospitalized injury population are interrelated, but primarily due to financial factors, physical limitations, and need for patient education. Solutions such as improving discharge planning, implementing voucher programs or free or low-cost transport vehicles warrant further study to determine feasibility and cost-effectiveness.

## Supporting information

S1 Data KCMC clinical trauma registry. (DOCX)

S2 Data Two-week survey tool. (DOCX)

S1 Text Community advisory board focus group script. (DOCX)

S2 Text Family interview script. (PDF)
