# Supplementary material for: Barriers to accessing follow up care in post-hospitalized trauma patients in Moshi, Tanzania: A mixed methods study
Source: PLOS Glob Public Health. 2022 Jun 13;2(6):e0000277. doi: 10.1371/journal.pgph.0000277 (PMC10021180; doi:10.1371/journal.pgph.0000277)
Supplement: S1 Data — (DOCX) [file pgph.0000277.s001.docx]

**DEMOGRAPHICS**

Where patient lives: ☐ Moshi Urban ☐Moshi Rural

☐ Other: ____________________

Patient age: ______

Patient sex: ☐Male ☐Female

Patient Years Education: ______

Marital status: ☐ Single ☐ Married ☐ Partner, not married

☐ Widow/Widower ☐ Separated

Employment: ☐ Student ☐ Unemployed

☐ Professional ☐ Skilled employment ☐ Self-employed ☐ Farmer ☐Other:_______________________

Tribe: ☐ Chagga ☐ Sambaa ☐ Masai ☐ Pare ☐ Sukuma ☐ Iraq ☐ Nyaturu ☐ Mmeru

☐ Muha ☐ Other: ______________

Type of insurance:

☐ None/ abscond

☐ Cash personal payment/relative support

☐ National Health Insurance

☐ Hospital support/exemption *(determined at discharge)*

☐ Other: _______________________

**MEDICAL HISTORY**

Diabetes Mellitus:

☐No → ☐ Never tested ☐ Tested and Negative

☐Yes → ☐Untreated/Doesn’t follow up ☐Pills ☐ Insulin

Hypertension:

☐No → ☐Never tested ☐Tested and Negative

☐Yes → ☐Takes pills ☐Treated but uncontrolled

☐Untreated or Doesn’t follow up

Any prior surgery:

☐No

☐ Yes→ ☐Appendectomy ☐Cholecystectomy

☐ Other:______________________

☐ Other: ______________________

Any prior TBI?: ☐No ☐ Yes

HIV Status: ☐ Tested and negative

☐ Never tested

☐ Yes ↓

*ARTs?* ☐*No* ☐*Yes → Started (mm/yyyy) _ _ /_ _ _ _*

*On Septrin?* ☐ *No* ☐ *Yes*

*Date of 1st pos. test (mm/yyyy): _ _ / _ _ _ _*

*Last Viral Load:______ Last CD4 count: ________*

*Clinic:___________________________________*

*Any AIDS related infections:__________________*

*_______________________________________*

**Prescribed Medications:** (medication name, dose, frequency)

☐_______________________________________________ ☐___________________________________________________

☐_______________________________________________ ☐___________________________________________________

☐_______________________________________________ ☐___________________________________________________

☐_______________________________________________ ☐___________________________________________________

**Demographics & History: ☐ patient reported ☐ family reported**

**BEHAVIORAL HISTORY ON ARRIVAL; SF8 SF8 ☐ patient reported ☐ family reported**

| **SF0. Kwa kutumia mstari kama kielelezo, unawezaje kupima/kukadiria vipi hali ya afya yako kwa sasa? *(Nakili kwa asilimia inayoendana na alama ya anayehojiwa)*** From 0 to 100, how would you rate your current state of health? (Record number): __________  You can use the line as a guide: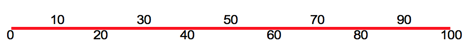 | | | | | | |
| --- | --- | --- | --- | --- | --- | --- |
| **SF1.** **Kwa ujumla, unawezaje kukadiria afya yako kwa wiki 4 zilizopita?**  Overall, how would you rate your health during the past **4 weeks**? | *0: Bora Zaidi*  0: Excellent | *1: Nzuri sana*  *1: Very Good* | *2: Nzuri*  *2: Good* | *3: Inaridhisha*  *3: Fair* | *4: Duni*  *4: Poor* | *5:Hairidhishi*  *5: Very Poor* |
| **SF2. Kwa wiki 4 zilizopita, ni kiasi gani matatizo yako ya kiafya yalizuia shughuli zako za kawaida za kimwili (kama vile kutembea kwenda sokoni)?**  During the past **4 weeks**, how much did physical health problems limit your usual physical activities (such as walking to the market)? | *0: Hapana kabisa*  *0: Not at all* | *1: Kidogo sana*  *1: Very little* | *2: Kiasi*  *2: Somewhat* | *3: Sana*  *3: Quite a lot* | *4: Sikuwa naweza kufanya shughuli za kimwili*  *4: Could not do physical activities* | |
| **SF3. Katika wiki 4 zilizopita, ulipata ugumu kwa kiasi gani katika kufanya shughuli zako za kila siku, ukiwa nyumbani au mbali na nyumbani , kwa sababu ya afya yako ya kimwili?**  During the past **4 weeks**, how much difficulty did you have doing your daily work, both home and away from home, because of your physical health? | *0: Hapana kabisa*  *0: None at all* | *1: Kidogo sana*  *1: A little bit* | *2: Kiasi*  *2: Some* | *3: Sana*  *3: Quite a lot* | *4: Sikuweza kufanya shughuli kilasiku*  *4: Could not do daily work* | |
| **SF4 Umekuwa na maumivu ya mwili kiasi gani kwa wiki 4 zilizopita?**  How much bodily pain have you had during the past **4 weeks**? | *0: Hakuna*  *0: None* | *1: Kidogo sana*  *1: Very mild* | *2: Kidogo*  *2: Mild* | *3: Kiasi*  *3:Moderate* | *4:Makal*  *4:Severe* | *5:Makali sana*  *5: Very Severe* |
| **SF5. Kwa wiki 4 zilizopita, ulikuwa na nguvu kiasi gani?**  During the past **4 weeks**, how much energy did you have? | *0: Nyingi sana*  *0: Very much* | *1:Nyingi zaidi*  *1: Quite a lot* | *2:Kaisi*  *2: Some* | *3: Kidogo*  *3: A little* | *4:Hakuna*  *4: None* | |
| **SF6.** **Kwa wiki 4 zilizopita ni kwa kiasi gani hali yako ya afya ya kimwili au matatizo ya kihisia yalizuia shughuli zako za kijamii na familia au marafiki?**  During the past **4 weeks**, how much did your physical health or emotional problems limit your usual social activities with family or friends? | *0: Hapana kabisa*  *0: None at all* | *1: Kidogo sana*  *1: Very little* | *2: Kidogo*  *2: Somewhat* | *3: Nyingi sana*  *3: Quite a lot* | *4: Sikuwa naweza kufanya shughuli za kijamii*  *4: Could not do social activities* | |
| **SF7.** **Kwa wiki 4 zilizopita, umesumbuliwa kwa kiasi gani na shida za mhemko (kama vile kujihisi kuwa na wasiwasi, mfadhaiko au kuwashwa)?**  During the past **4 weeks**, how much have you been bothered by emotional problems (such as feeling anxious, depressed or irritable)? | *0: Hapana kabisa*  *0: None at all* | *1: Kidogo*  *1: Slightly* | *2:Kiasi*  *2:Moderately* | *3:Nyingi sana*  *3: Quite a lot* | *4:Kuzidi kiasi*  *4: Extremely* | |
| **SF8. Kwa wiki 4 zilizopita ni kwa kiasi gani matatizo binafsi au ya hisia yalikuzuia kufanya kazi zako za kawaida, shughuli za shule au shughuli nyiingine za kila siku?**  During the past **4 weeks**, how much did personal or emotional problems keep you from doing your usual work, school or other daily activities? | *0: Hapana kabisa*  *0: None at all* | *1: Kidogo*  *1: Slightly* | *2: Kiasi*  *2: Moderately* | *3: Nyingi sana*  *3:Quite a lot* | *4: Kuzidi kiasi*  *4: Extremely* | |

**PHQ-2 DEPRESSION PHQ2 ☐ patient reported ☐ family reported**

| **PHQ1. Kwa wiki 2 ziilizopita ni kwa kiasi gani umekuwa ukisumbuliwa na kutokuwa na hamu au shauku ya kufanya vitu:**  In the past two weeks how often have you been bothered by little interest or pleasure in doing things: | *0: Hapana kabisa*  *0: Not at all* | *1:Siku kadhaa*  *1: Several days* | *2: Zaidi ya nusu ya siku zote*  *2: More than half the days* | *3: Karibu kila siku*  *3: Nearly every day* |
| --- | --- | --- | --- | --- |
| **PHQ2. Kwa wiki 2 zilizopita ni kwa kiasi gani umekuwa ukisumbuliwa na kuzubaa,kufadhaika au kukosa matumaini:**  In the past two weeks how often have you been bothered by feeling down, depressed, or hopeless: | *0: Hapana kabisa*  *0: Not at all* | *1:Siku kadhaa*  *1: Several days* | *2: Zaidi ya nusu ya siku zote*  *2: More than half the days* | *3: Karibu kila siku*  *3: Nearly every day* |

**AUDIT Alcohol consumption**: “Now I am going to ask you some questions about your use of alcoholic beverages during this past year.” Explain what is meant by “alcoholic beverages” by using local examples of beer, wine, vodka, etc.

**AUDIT ☐ patient reported ☐ family reported**

| **1. Mara ngapi unatumia kinywaji kilicho na kilevi?**  How often do you have a drink containing alcohol? | *Hakuna*  *Never* | *Kila mwezi au chini ya mwezi*  *Monthly or less* | *Mara 2 hadi 4 kwa mwezi*  *2-4 times/month* | *Mara 2 hadi 3 kwa wiki*  *2-3 times / week* | *4 au zaidi kwa wiki*  *4 or more times/week* |
| --- | --- | --- | --- | --- | --- |
| **2. Kwa siku ya kawaidia unatumia vinywaji vingapi vyenye kilevi unapokuwa unakunywa?**  How many drinks containing alcohol do you have on a typical day when you are drinking? | *1 au 2* | *3 au 4* | *5 au 6* | *7 au 9* | *10 au zaidi* |
| **3. Mara ngapi unatumia vinywaji sita au zaidi kwa mara moja?**  How often do you have six or more drinks on one occasion? | *Haijawahi kutokea*  *Never* | *Chini ya kila mwezi*  *Less than monthly* | *Kila Mwezi*  *Monthly* | *Kwa wiki*  *Weekly* | *Kila siku au karibu kila siku*  *Daily/ almost daily* |
| **4. Mara ngapi katika mwaka uliopita uligundua hukuweza kuacha kunywa mara ukishaanza?**  How often during the **last year** have you found that you were not able to stop drinking once you had started? | *Haijawahi kutokea*  *Never* | *Chini ya kila mwezi*  *Less than monthly* | *Kila Mwezi*  *Monthly* | *Kwa wiki*  *Weekly* | *Kila siku au karibu kila siku*  *Daily/ almost daily* |
| **5. Mara ngapi katika mwaka uliopita ulishindwa kufanya unavyotarajiwa kutoka kwako kwa sababu ya kunywa?**  How often during the **last year** have you failed to do what was normally expected of you because of drinking? | *Haijawahi kutokea*  *Never* | *Chini ya kila mwezi*  *Less than monthly* | *Kila Mwezi*  *Monthly* | *Kwa wiki*  *Weekly* | *Kila siku au karibu kila siku*  *Daily/ almost daily* |
| **6. Mara ngapi katika mwaka uliopita ulihitaji kinywaji cha kwanza asubuhi ili kuweza kuendelea na shughuli zako baada ya kunywa sana?**  How often during the **last year** have you needed a first drink in the morning to get yourself going after a heavy drinking session? | *Haijawahi kutokea*  *Never* | *Chini ya kila mwezi*  *Less than monthly* | *Kila Mwezi*  *Monthly* | *Kwa wiki*  *Weekly* | *Kila siku au karibu kila siku*  *Daily/ almost daily* |
| **7. Mara ngapi katika mwaka uliopita ulijihisi kuwa na hatia au kujilaumu baada ya kunywa?**  How often during the **last year** have you had a feeling of guilt or remorse after drinking? | *Haijawahi kutokea*  *Never* | *Chini ya kila mwezi*  *Less than monthly* | *Kila Mwezi*  *Monthly* | *Kwa wiki*  *Weekly* | *Kila siku au karibu kila siku*  *Daily/ almost daily* |
| **8. Mara ngapi katika mwaka uliopita hukuweza kukumbuka kilichotendeka usiku uliopita kwa sababu ulikunywa?**  How often during the **last year** have you been unable to remember what happened the night before because of your drinking? | *Haijawahi kutokea*  *Never* | *Chini ya kila mwezi*  *Less than monthly* | *Kila Mwezi*  *Monthly* | *Kwa wiki*  *Weekly* | *Kila siku au karibu kila siku*  *Daily/ almost daily* |
| **9. Je, umejeruhiwa au mtu mwingine kujeruhiwa kwa sababu ya kunywa kwako?**  Have you or someone else been injured because of your drinking? | *Hapana [0]*  *No [0]* |  | *Ndiyo, lakini ki kwa mwaka uliopita [2]*  *Yes, but not in the last year [2]* |  | *Ndiyo kwa mwaka uliopita [4]*  *Yes, during the last year [4]* |
| **10. Je, ndugu yako au rafiki yako au daktari au mhudumu wa afya mwingine ameguswa na kunywa kwako au kupendekeza upunguze kunywa kwako?**  Has a relative, friend, doctor, or other health care worker been concerned about your drinking or suggested you cut down? | *Hapana [0]*  *No [0]* |  | *Ndiyo, lakini ki kwa mwaka uliopita [2]*  *Yes, but not in the last year [2]* |  | *Ndiyo kwa mwaka uliopita [4]*  *Yes, during the last year [4]* |

**SUBSTANCE USE: ☐ patient reported ☐ family reported**

| **Drug** | **Lifetime Use?** | **Current Use?** | **Quantity per week** |
| --- | --- | --- | --- |
| Tobacco | ☐No ☐Yes→ | ☐No ☐Yes→ |  |
| Marijuana | ☐No ☐Yes→ | ☐No ☐Yes→ |  |
| Cocaine | ☐No ☐Yes→ | ☐No ☐Yes→ |  |
| Heroin | ☐No ☐Yes→ | ☐No ☐Yes→ |  |
| Mirungi (Khat) | ☐No ☐Yes→ | ☐No ☐Yes→ |  |
| Tambu | ☐No ☐Yes→ | ☐No ☐Yes→ |  |
| Kuberi (oral snuff) | ☐No ☐Yes→ | ☐No ☐Yes→ |  |

**ACUTE INJURY INFORMATION:**

Date of Injury: (dd/mm/20yy) __ __/__ __/20__ __

Injury time: (24 hr) __ __:__ __

Date of Arrival to KCMC: (dd/mm/20yy) ____/____/20____

Arrival time: (24 hr) __ __:__ __

**Mechanism of arrival:** ☐ Ambulance from other hospital

☐ Private car ☐ Bajaji ☐ Police car

☐ Private Motorcycle ☐ Boda boda ☐ Taxi

☐ Unknown ☐ Other: ___________________

**First health center treated at:**

☐ KCMC ☐ Hai District Hosp. ☐ Same District Hosp.

☐ Kilema Hosp. ☐ Kibosho Hosp. ☐ Faraja Hosp.

☐ Mawenzi ☐ Siha District Hosp. ☐ St. Joseph Hosp.

☐ Other:_______________________

Arrival date: (dd/mm/yy) __ __/__ __/__ __

Arrival time: (24 hr) __ __:__ __

**Additional health center treated at:**

☐ NONE ☐ Hai District Hosp. ☐ Same District Hosp.

☐ Kilema Hosp. ☐ Kibosho Hosp. ☐ Faraja Hosp.

☐ Mawenzi ☐ Siha District Hosp. ☐ St. Joseph Hosp.

☐ Other:_______________________

Arrival date: (dd/mm/yy) __ __/__ __/__ __

Arrival time: (24 hr) __ __:__ __

**Intention of the Injury**:

☐ Unknown

☐ Unintentional

☐ Intentional→ ☐ Self-Inflicted

☐ Inflicted by other person

☐ Inflicted by other (non-person)

**Mechanism of Injury:**

☐ Unknown

☐ Road Traffic→

☐ Driver or ☐Passenger

*Vehicle type→*  ☐ *Motorcycle* ☐ *Truck*

☐ *Car* ☐ *Dala dala*

☐ *Bajaji* ☐ *Bus*

☐ Pedestrian

☐ Bicycle

☐ Assault→ ☐ Fist/Foot ☐ Gun ☐ Knife ☐Other:_________

☐ Drowning

☐ Fall→ ☐ Fall from standing

☐ Fall from height: _______meters from ground

☐ Burn

☐ Other:_______________________________________

**Alcohol Status:**

Patient: ☐ Negative ☐ Positive

How was information obtained? ☐ Breathalyzer: _________

☐ Clinical Exam ☐ History

Driver, if not patient: ☐ N/A ☐ Negative ☐ Unknown

☐ Positive → ☐ Suspected

☐ Confirmed

Other persons involved: ☐ N/A ☐ Negative ☐ Unknown

☐ Positive → ☐ Suspected

☐ Confirmed

**BASELINE RISK ASSESSMENT:
Vital Signs on Arrival to ED:**

T _____ RR ______ HR ______ BP ______ / ______

Pulse Ox ______ Pain Level (0-100)_______ MUAC: _____ cm Height_____cm Weight______kg AVPU:_____

Date:(dd/mm/20yy) ____/____/20____ Time: (24 hr) __ __:__ __

**AVPU** *(Choose one)*

☐Alert ☐Responds to Verbal stimuli only

☐Unresponsive ☐Responds to Painful Stimuli only

**Pupils:** L: ☐normal ☐sluggish ☐ non-reactive ☐ untestable

R: ☐normal ☐sluggish ☐ non-reactive ☐ untestable

**Are pupils even?** ☐ No ☐ Yes

**Was this injury a TBI?**  ☐ No ☐ Yes

| **Eye Opening**  (choose one) | *Spontaneously* | *4* |
| --- | --- | --- |
|  | *To Speech* | *3* |
|  | *To Pain* | *2* |
|  | ***None*** | ***1*** |
| **Verbal Response** (choose one) | *Oriented* | *5* |
|  | *Confused* | *4* |
|  | *Inappropriate* | *3* |
|  | *Incomprehensible* | *2* |
|  | ***None*** | ***1*** |
| **Motor Response** (choose one) | *Obeys Commands* | *6* |
|  | *Localizes to pain* | *5* |
|  | *Withdraws from pain* | *4* |
|  | *Flexion to pain* | *3* |
|  | *Extension to pain* | *2* |
|  | ***None*** | ***1*** |

**Secondary insults and other complications:**

Did patient experience seizure?

☐ No

☐ Yes→ Type:_______ Duration: ______(minutes)

Any aspiration event? ☐ No ☐ Yes

Burn? ☐ No ☐ Yes →

If Yes, Total BSA% Burned:__________

**HOSPITAL STATUS**

**Vital Signs on Leaving ED**/Arrival to ward/ICU**:**

T _____ RR ______ HR ______ BP ______ / ______

Pulse Ox ______ Pain Level (0-100)_______ MUAC: _____ cm Height_____cm Weight______kg

AVPU:_____ GCS E___/ V____/ M____

Date:(dd/mm/20yy) ____/____/20____ Time: (24 hr) __ __:__ __

**ED Dispo location**: ☐ Theatre/OR ☐ ICU ☐ Surgical 1

☐ Surgical 2 ☐ SubICU

☐ Other:______________________

**Did the patient’s status worsen after the ED?** ☐ No ☐ Yes

**ICU?** ☐ No (proceed to “SubICU?”) ☐ Yes (continue)

Date of Arrival to ICU (dd/mm/20yy): __ __ / __ __ /20__ __ Time: (24 hr) __ __:__ __

Vital Signs on **Arriving** to ICU**:**

T _____ RR ______ HR ______ BP ______ / ______ Pulse Ox ______ Pain Level (0-100)_______

MUAC: _____ cm Height_____cm Weight______kg AVPU:_____ GCS E___/ V____/ M____

Date of Discharge from ICU (dd/mm/yy):__ __ / __ __ /20__ __ Time: (24 hr) __ __:__ __

Vital Signs on **Leaving** ICU**:**

T _____ RR ______ HR ______ BP ______ / ______ Pulse Ox ______ Pain Level (0-100)_______

MUAC: _____ cm Height_____cm Weight______kg AVPU:_____ GCS E___/ V____/ M___

**SubICU?** ☐ No (proceed to “Needed intubation?”) ☐ Yes (continue)

Date of Arrival to SubICU (dd/mm/20yy): __ __ / __ __ /20__ __ Time: (24 hr) __ __:__ __

Vital Signs on **Arriving** to SubICU**:**

T _____ RR ______ HR ______ BP ______ / ______ Pulse Ox ______ Pain Level (0-100)_______

MUAC: _____ cm Height_____cm Weight______kg AVPU:_____ GCS E___/ V____/ M____

Date of Discharge from SubICU (dd/mm/yy):__ __ / __ __ /20__ __ Time: (24 hr) __ __:__ __

Vital Signs on **Leaving** SubICU**:**

T _____ RR ______ HR ______ BP ______ / ______ Pulse Ox ______ Pain Level (0-100)_______

MUAC: _____ cm Height_____cm Weight______kg AVPU:_____ GCS E___/ V____/ M___

**Needed intubation?** ☐ No ☐ Yes→ If yes, where?_______________________________

→ Intubation: (dd/mm/20yy):__ __ / __ __ /20__ __ Time: (24 hr) __ __:__ __

Extubation: (dd/mm/20yy) ___/___/20___ Time: (24 hr) ___:___

**ORU?** ☐ No ☐ Yes **→ Date to ORU:** (dd/mm/yy) ___/___/___

**Nutrition information and status/risks**

Difficulty eating during hospitalization? ☐ No ☐ Yes

NGT placed for nutrition: ☐ No ☐ Yes

Date nutrition started: (dd/mm/20yy):____ / ____ /20___

**DIAGNOSTICS INFORMATION:**

POCUS/ EFAST: ☐ No ☐ Yes→ Date (dd/mm/yy) ___/___/___ Time: (24 hr) ___:___

RUQ: ☐ Normal ☐ Not performed ☐ Abnormal Findings:____________________

LUQ: ☐ Normal ☐ Not performed ☐ Abnormal Findings:____________________

Cardiac: ☐ Normal ☐ Not performed ☐ Abnormal Findings:____________________

Pelvis: ☐ Normal ☐ Not performed ☐ Abnormal Findings:____________________

Lung: ☐ Normal ☐ Not performed ☐ Abnormal Findings:____________________

HGb tested? ☐ No ☐ Yes initial:_______, lowest hgb:_____

Needed a transfusion? ☐ No ☐ Yes: Date (dd/mm/yy) ___/___/___

Glucose tested? ☐ No ☐ Yes_________

Creatinine tested? ☐ No ☐ Yes First_______Date (dd/mm/yy) ___/___/___

Highest Cre_____ Date (dd/mm/yy) ___/___/___

**XR Obtained**: ☐ No ☐ Yes

Date: (dd/mm/20yy):__ __ / __ __ /20____ Time: (24 hr) __ __:__ __

XRay Results:

☐ _________________________________________ ☐ ___________________________________________

☐ _________________________________________ ☐ ___________________________________________

**CT obtained:** ☐ No ☐ Yes

Date: (dd/mm/20yy):__ __ / __ __ /20____ Time: (24 hr) __ __:__ __

CT Results:

☐ _________________________________________ ☐ ___________________________________________

☐ _________________________________________ ☐ ___________________________________________

Subarachnoid Hemorrhage:☐ Absent ☐ Present ☐ Indeterminant ☐N/A

Subdural Hemorrhage: ☐ Absent ☐ Present ☐ Indeterminant ☐N/A

Epidural Hemorrhage: ☐ Absent ☐ Present ☐ Indeterminant ☐N/A

**PERIOPERATIVE/ OPERATIVE DATA**

**Needed surgery?**

**Surgery#1**:☐ No ☐ Yes→ date(dd/mm/20yy):__ __ / __ __ /20__ __ Time: (24 hr) __ __:__ __

Indication:____________________________________________ WHO Checklist: ☐ No ☐ Yes

Procedure:___________________________________________ ASA Status: ☐ I ☐ II ☐ III ☐ IV ☐ V ☐Unknown

**Surgery Type**: ☐ Orthopedic ☐ General ☐ Neurosurgery ☐ ENT/Otolaryngology) ☐ Other

**Anesthesia:**  ☐General ☐Spinal/Epidural ☐Local *(see below)*

**OR Airway Mgmt:** ☐ NA/none ☐ NRB ☐ Mask Ventilation ☐ LMA ☐ Intubation

**Induction:** ☐NA ☐ Ketamine ☐Thiopental ☐ Propofol ☐ Benzodiazepine ☐ Lignocaine ☐ Bupivacaine

**Analgesia:** ☐NA ☐Fentanyl ☐ Morphine ☐ Tramadol ☐ Pethadine ☐ Paracetamol ☐NSAID

**Paralytic:** ☐NA ☐Succinylcholine

**Nerve block**: ☐ No ☐Yes → **US guided?** ☐No ☐ Yes

**Type**: ☐Fascia iliaca ☐Femoral ☐Adductor canal ☐Popliteal ☐Caudal

☐TAP ☐Rectus sheath ☐Ilioinguinal ☐Interscalene ☐Axillary

☐PEC serratus anterior ☐Supraclavicular ☐Infraclavicular

**Post-op nausea/vomiting medications**: ☐ No ☐Yes → Which:______________________________________

**Surgery date#2:**☐ No ☐ Yes (dd/mm/20yy):__ __ / __ __ /20__ Time: (24 hr) __ __:__ __

Indication:____________________________________________ WHO Checklist: ☐ No ☐ Yes

Procedure:___________________________________________ ASA Status: ☐ I ☐ II ☐ III ☐ IV ☐ V ☐Unknown

**Surgery Type**: ☐ Orthopedic ☐ General ☐ Neurosurgery ☐ ENT/Otolaryngology) ☐ Other

**Anesthesia:**  ☐General ☐Spinal/Epidural ☐Local *(see below)*

**OR Airway Mgmt:** ☐ NA/none ☐ NRB ☐ Mask Ventilation ☐ LMA ☐ Intubation

**Induction:** ☐NA ☐ Ketamine ☐Thiopental ☐ Propofol ☐ Benzodiazepine ☐ Lignocaine ☐ Bupivacaine

**Analgesia:** ☐NA ☐Fentanyl ☐ Morphine ☐ Tramadol ☐ Pethadine ☐ Paracetamol ☐NSAID

**Paralytic:** ☐NA ☐Succinylcholine

**Nerve block**: ☐ No ☐Yes → **US guided?** ☐No ☐ Yes

**Type**: ☐Fascia iliaca ☐Femoral ☐Adductor canal ☐Popliteal ☐Caudal

☐TAP ☐Rectus sheath ☐Ilioinguinal ☐Interscalene ☐Axillary

☐PEC serratus anterior ☐Supraclavicular ☐Infraclavicular

**Post-op nausea/vomiting medications**: ☐ No ☐Yes → Which:______________________________________

**Surgery date#3:**☐ No ☐ Yes (dd/mm/20yy):__ __ / __ __ /20___ Time: (24 hr) __ __:__ __

Indication:____________________________________________ WHO Checklist: ☐ No ☐ Yes

Procedure:___________________________________________ ASA Status: ☐ I ☐ II ☐ III ☐ IV ☐ V ☐Unknown

**Surgery Type**: ☐ Orthopedic ☐ General ☐ Neurosurgery ☐ ENT/Otolaryngology) ☐ Other

**Anesthesia:**  ☐General ☐Spinal/Epidural ☐Local *(see below)*

**OR Airway Mgmt:** ☐ NA/none ☐ NRB ☐ Mask Ventilation ☐ LMA ☐ Intubation

**Induction:** ☐NA ☐ Ketamine ☐Thiopental ☐ Propofol ☐ Benzodiazepine ☐ Lignocaine ☐ Bupivacaine

**Analgesia:** ☐NA ☐Fentanyl ☐ Morphine ☐ Tramadol ☐ Pethadine ☐ Paracetamol ☐NSAID

**Paralytic:** ☐NA ☐Succinylcholine

**Nerve block**: ☐ No ☐Yes → **US guided?** ☐No ☐ Yes

**Type**: ☐Fascia iliaca ☐Femoral ☐Adductor canal ☐Popliteal ☐Caudal

☐TAP ☐Rectus sheath ☐Ilioinguinal ☐Interscalene ☐Axillary

☐PEC serratus anterior ☐Supraclavicular ☐Infraclavicular

**Post-op nausea/vomiting medications**: ☐ No ☐Yes → Which:______________________________________

**ICU Risk factors: Check all that apply:** ☐ Never in ICU ☐ None apply ☐ aspiration event/ head not 30 deg

☐ Hypoxia ☐ Hyperglycemia ☐ Hypotension

☐ Hgb<6 for 72 hours (no transfusion for 72hrs)

**Did pt get GI Ppx**(Ranitidine, Cimetidine, Omeprazole, Lantoprazole)**?** ☐ No ☐ Yes → Onset Date: (dd/mm/20yy):__ __ / __ __ /20____

**Did pt get DVT ppx**(Heparin, Aspirin, Enoxaparin) ☐ No ☐Yes→ Onset Date: (dd/mm/20yy):__ __ / __ __ /20____

**Did pt first get rehab in the ICU?**  ☐ No ☐Yes→ Onset Date: (dd/mm/20yy):__ __ / __ __ /20____

**Did pt get seizure medicines?** ☐ No ☐Yes→ Onset Date: (dd/mm/20yy):__ __ / __ __ /20____

→ Which? ☐ Phenytoin ☐ Carbamazepine ☐ other____________

**Did pt get antibiotics during hospitalization?** ☐ No ☐Yes→ If yes, which?

☐ Ampicillin # days___ ☐ Gentamicin # days___ ☐ Ceftriaxone # days___

☐Metronidazole # days___ ☐Piperacillin/Tazobactam # days___ ☐ other____________ # days___

**Diagnostics List: (Clinical Impression)**

1. ________________________________________________________________________________________
2. ________________________________________________________________________________________
3. ________________________________________________________________________________________
4. ________________________________________________________________________________________
5. ________________________________________________________________________________________
6. ________________________________________________________________________________________
7. ________________________________________________________________________________________
8. ________________________________________________________________________________________
9. ________________________________________________________________________________________

**Complications**

| Pneumonia | ☐ No ☐Yes→ | ☐Aspiration ☐ Ventilator Associated ☐Other |
| --- | --- | --- |
| Acute renal Injury | ☐ No ☐Yes |  |
| Thrombosis | ☐ No ☐Yes→ | If yes: ☐DVT ☐PE ☐Fat embolism  If yes: ☐ Clinical dx ☐ US ☐ CT |
| Urinary tract infection | ☐ No ☐Yes |  |
| Multiple organ failure secondary to sepsis and shock | ☐ No ☐Yes |  |
| Unexpected Delirium | ☐ No ☐Yes |  |
| Sympathetic Storm | ☐ No ☐Yes | *[tachypnea, diaphoresis, hypertensive post TBI]* |
| Seizures | ☐ No ☐Yes |  |
| Wound infections | ☐ No ☐Yes |  |
| Wound dehiscence/rupture/burst | ☐ No ☐Yes |  |
| Decubitus Ulcer/ Bed Sore | ☐ No ☐Yes→ | If yes: ☐Stage 1 ☐Stage 2 ☐Stage 3 ☐Stage 4 |

**Providers involved in care:**

**PT/OT** ☐ No ☐Yes → Date of first service: (dd/mm/yy) ____/____/_____ Time: (24 hr) ____:____

**Medicine Consultation** ☐ No ☐Yes→ for: _______________________________________

**Cardiology Consultation** ☐ No ☐Yes→ for: _____________________________________

**Social Worker** ☐ No ☐Yes→ for: _______________________________________

**Other:** ___________________________ for: ______________________________________

**SURGICAL DISCHARGE DATA:**

**Discharge Vital Signs: Maumivu yako wapi?** (Where is your pain?) Pain (0-100)_______

T _____ RR ______ HR ______ BP ______ / ______
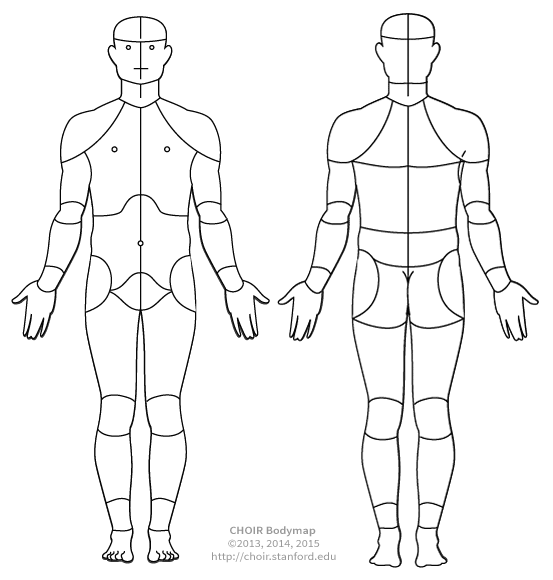


Pulse Ox _______ Weight______kg Height_____cm

MUAC: _____ cm AVPU:______ GCS E____/ V_____/ M____

Date:(dd/mm/20yy) ____/____/20____ Time: (24 hr) __ __:__ __

**Discharge from hospital:** ☐ No (Elope/Death) ☐ Yes

Date: (dd/mm/yy) ___/___/___

Time: (24 hr) __ __:__ __

**Death in the hospital:** ☐ No ☐ Yes

Date: (dd/mm/yy) ___/___/___

Time: (24 hr) __ __:__ __

**Destination** after discharge: ☐ Morgue ☐ Home

☐ Other:_____________

**In-hospital meds:**

| **Prescribed Medication Name** | **Dose** | **Frequency** | **Start Date** |
| --- | --- | --- | --- |
| ☐1 |  | ☐ ___ times daily ☐ as needed ☐ one-time/stat |  |
| ☐2 |  | ☐ ___ times daily ☐ as needed ☐ one-time/stat |  |
| ☐3 |  | ☐ ___ times daily ☐ as needed ☐ one-time/stat |  |
| ☐4 |  | ☐ ___ times daily ☐ as needed ☐ one-time/stat |  |
| ☐5 |  | ☐ ___ times daily ☐ as needed ☐ one-time/stat |  |
| ☐6 |  | ☐ ___ times daily ☐ as needed ☐ one-time/stat |  |

**Discharge meds and devices:**

| **Prescribed Medication or Device Name** | **Dose** | **Frequency** | **New?** *(since hospitalization)* |
| --- | --- | --- | --- |
| ☐1 |  | ☐ ___ times daily ☐ as needed | ☐ Yes ☐ No |
| ☐2 |  | ☐ ___ times daily ☐ as needed | ☐ Yes ☐ No |
| ☐3 |  | ☐ ___ times daily ☐ as needed | ☐ Yes ☐ No |
| ☐4 |  | ☐ ___ times daily ☐ as needed | ☐ Yes ☐ No |
| ☐5 |  | ☐ ___ times daily ☐ as needed | ☐ Yes ☐ No |
| ☐6 |  | ☐ ___ times daily ☐ as needed | ☐ Yes ☐ No |

**Follow-up Plans:**

| **Comorbidities** | **Surgery** ☐No ☐Yes | **Dx:** | **When:** |
| --- | --- | --- | --- |
|  | **Orthopedics** ☐No ☐Yes |  |  |
|  | **Medicine** ☐No ☐Yes |  |  |
|  | **Medicine #2** ☐No ☐Yes |  |  |
|  | **Medicine #3** ☐No ☐Yes |  |  |
|  | **Medicine #4** ☐No ☐Yes |  |  |
| **Rehab/OT, Pain** | **Rehab** ☐No ☐Yes |  |  |
|  | **OT** ☐No ☐Yes |  |  |
|  | **Other:____________** ☐No ☐Yes |  |  |
| **Mental Health** | **_________________** ☐No ☐Yes |  |  |
| **Substance Use** | **_________________** ☐No ☐Yes |  |  |

**Glasgow Outcomes Scale at discharge**

| 8 | Upper good recovery, GR+ | Resumption of normal life despite minor deficits |
| --- | --- | --- |
| 7 | Lower good recovery, GR- | Nearly resuming normal life |
| 6 | Upper moderate disability, MD+ | Disabled but independent. Can work in a sheltered setting. Needs minor modification. |
| 5 | Lower moderate disability | Unable to fully function outside home (work, shopping, social activities, psych.) |
| 4 | Upper severe disability, SD+ | Conscious but disabled. Dependent for daily support. Needs assistance outside home |
| 3 | Lower severe disability, SD- | Need frequent support at home |
| 2 | Persistent vegetative, VS | Minimal responsiveness, unconscious, cannot speak or obey commands |
| 1 | Death | Non survival |

**PHQ2 at discharge: ☐ patient reported ☐ family reported**

| **PHQ 1. Kwa wiki 2 ziilizopita ni kwa kiasi gani umekuwa ukisumbuliwa na kutokuwa na hamu au shauku ya kufanya vitu?**  *In the past two weeks how often have you been bothered by little interest or pleasure in doing things:* | *0: Hapana kibisa*  *Not at all* | *1: Siku kadhaa*  *Several days* | *2: Zaidi ya nusu ya siku zote*  *More than half the days* | *3: Karibu kila siku*  *Nearly every day* |
| --- | --- | --- | --- | --- |
| **PHQ 2. Kwa wiki 2 zilizopita ni kwa kiasi gani umekuwa ukisumbuliwa na kuzubaa, kufadhaika au kukosa matumaini?**  *In the past two weeks how often have you been bothered by feeling down, depressd, or hopeless:* | *0: Hapana kibisa*  *Not at all* | *1: Siku kadhaa*  *Several days* | *2: Zaidi ya nusu ya siku zote*  *More than half the days* | *3: Karibu kila siku*  *Nearly every day* |

**Family Support Questions:**

Who would you call on for help if you were sick or disabled?

Primary caregiver’s name and relation:_______________________________________________

Would (person’s name) be available to you if you needed assistance after this hospitalization?

☐ No ☐Yes ☐ Don’t know ☐ No response

When would this person be available to you?

☐ Never ☐ Weekdays ☐ Weekends ☐Both ☐ Don’t know ☐ No response

What time of day would this person be available to you?

☐ Daytime ☐ Evening ☐ Overnight

**Functional Independence Measure at discharge ☐ patient reported ☐ family reported**

| FIM1. Self care | Score: | **1, Total assistance** (Subject contributes <25% of the effort or is unable to do the task)  **2, Maximal assistance** (Subject provides less than half of the effort (25-49%)  **3, Moderate assistance** (Subject still performs 50-75% of the task)  **4, Minimal assistance** (Requiring incidental hand-on help only (subject performs >75% of the task)  **5, Supervision** ( Requiring only standby assistance or verbal prompting or help with set-up)  **6, Modified Independence** (Requiring the use of a device but not physical help)  **7, Complete independence** (Fully independent) |
| --- | --- | --- |
| FIM6. Toileting |  |  |
| FIM10. Transfers: bed/chair/wheelchair |  |  |
| FIM14. Locomotion: walking/wheelchair |  |  |
| FIM15. Locomotion: stairs |  |  |
| FIM17. Expression |  |  |
| FIM18. Comprehension |  |  |
| FIM22. Social Interaction |  |  |
| FIM26. Problem Solving |  |  |
| FIM27. Memory |  |  |
