# Supplementary material for: Barriers to accessing follow up care in post-hospitalized trauma patients in Moshi, Tanzania: A mixed methods study
Source: PLOS Glob Public Health. 2022 Jun 13;2(6):e0000277. doi: 10.1371/journal.pgph.0000277 (PMC10021180; doi:10.1371/journal.pgph.0000277)
Supplement: S2 Data — (DOCX) [file pgph.0000277.s002.docx]

**KCMC Transitions of Care Project (400 Study):**

**FOLLOW UP DATA, 2 weeks post discharge from hospital**

**Registry Study ID #__________ ToC Study ID:** T#__ __ __ __

**Patient Initials: ______ Age: _______ Gender:_______ Interview Location:** ☐ **Home**

☐ **Hospital**

T1. **Je! Una uwezo wa kuendesha / kujisafirisha mwenyewe?** Are you able to drive/transport yourself?

☐No ☐ Yes→

T1a. If yes, with what?

☐Car ☐ Motorcycle ☐ Bicycle

☐ Bajaji ☐ Dala dala ☐ Large bus ☐ Walking

| **If interviewed at the HOSPITAL** | **If interviewed at HOME or by PHONE** |
| --- | --- |
| T2. **Ulisafirije kuja hapa kwa ajili ya hudhurio hili?**  *How did you travel here for this appointment?*  ☐Car ☐ Motorcycle ☐ Bicycle  ☐ Bajaji ☐ Dala dala ☐ Large bus ☐ Walking  T3. **Ni nani aliye kusaidia safari yako kwa ajili ya hudhurio hili au mahudhurio mengine?**  *Who helped with your travel for this appointment or others?*  ☐ No one ☐ Mother ☐ Father ☐ Sister ☐ Brother  ☐ Son ☐ Daughter ☐ Other Family ☐ Friend ☐ Neighbor  ☐ Other (T3a):____________________________________  T4. **Je ilikuwa ngumu kuja kwenye hudhurio hili au lingine?**  *Was it difficult coming to this or other appointments?* ☐ No ☐ Yes  T4a. **If yes, why?**  ☐Too much walking/ Inahitaji kutembea sana  ☐Finding transportation/ Kutafuta usafiri  ☐Find someone to take you/ kupata mtu wa kukupeleka?  ☐Plan the time of day/ kupanga muda wa mchana  ☐Find the money to get gas, transport or help/ kupata pesa ya mafuta, usafiri au msaad  ☐ Other:/ Nyingine: (T4b)___________________  T5. **Je! Lmekua ngumu zaidi kusafiri tangu ulipoumia?**  *Is travel more difficult since your injury?* ☐ No ☐ Yes  T5a.**Kama ndiyo, ni ngumu zaidi kwa sababu ya:**  *If yes, is it more challenging because of:*  ☐ Financial challenges/ Changamoto za kipesa  ☐ Logistical Challenges/ Changamoto za taratibu za usafirishaji  ☐ Other/Nyingine (T5b):________________________________  T5c. **Kama ndiyo, ni nini unachokifanya cha tofauti kwa ajili ya usafiri kwa sasa kuliko kabla ya kuumia kwako?**  *If yes, what do you do differently for transport now than before your injury? _____________________________________________ _____________________________________________* | Tn2. **Hukuweza kusafiri kwenda hospitali kwa ajili ya hudhurio hii. Ni nini kilichofanya kuwa ngumu?**  *You were not able to travel to the hospital for this appointment. What made it hard?*  ☐Too much walking/ Inahitaji kutembea sana  ☐Finding transportation/ Kutafuta usafiri  ☐Find someone to take you/ kupata mtu wa kukupeleka?  ☐Plan the time of day/ kupanga muda wa mchana  ☐Find the money to get gas, transport or help/ kupata pesa ya mafuta, usafiri au msaad  ☐ Other:/ Nyingine: (Tn2a):___________________  Tn3. **Je! Lmekua ngumu zaidi kusafiri tangu ulipoumia?**  *Is travel more difficult since your injury?* ☐ No ☐ Yes→  Tn3a. **Kama ndiyo, ni ngumu zaidi kwa sababu ya:**  *If yes, is it more challenging because of:*  ☐ Financial challenges/ Changamoto za kipesa  ☐ Logistical Challenges/ Changamoto za taratibu za usafirishaji  ☐Other/Nyingine(Tn3b):_________________________  Tn3c. **Kama ndio, ni nini unachokifanya cha tofauti kwa ajili ya usafiri kwa sasa kuliko kabla ya kuumia kwako?** *If yes, what do you do differently for transport now than before your injury?*  __________________________________________  __________________________________________ |

| **mMOS-SS**: **Kama unahitaji, ni mara ngapi mtu anakuwepo ...** If you need it, how often is someone available... | | | | | |
| --- | --- | --- | --- | --- | --- |
|  |  |  |  |  |  |
| **SS1**. ...**Kukusaidia ikiwa huwezi kusogea kwa urahisi kama ilivyokuwa kabla ya kuumia**  SS1 ...To help you if you were unable to move as easily as before the injury | ***1: Haijawahi kutokea***  *1: None of the time* | ***2: Mara chache***  *2: A little of the time* | ***3: Baadhi ya muda***  *3: Some of the time* | ***4: Mara nyingi***  *4: Most of the time* | ***5: Mara zote***  *5: All of the time* |
| **SS2. ... Kukupeleka kwa daktari wakati unahitaji**  SS2. ...To take you to the doctor when you need it | ***1: Haijawahi kutokea*** | ***2: Mara chache*** | ***3: Baadhi ya muda*** | ***4: Mara nyingi*** | ***5: Mara zote*** |
| **SS3. ... Kuandaa chakula chako kama huwezi kufanya hivyo mwenyewe**  SS3. ...To prepare your meals if you were unable to do it yourself | ***1: Haijawahi kutokea*** | ***2: Mara chache*** | ***3: Baadhi ya muda*** | ***4: Mara nyingi*** | ***5: Mara zote*** |
| **SS4. ... Ili kukusaidia na kazi za kila siku na mambo ya ndani ya nyumba ikiwa huwezi kufanya hivyo mwenyewe**  SS4. ...To help you with daily chores and things in the house if you couldn’t do it for yourself | ***1: Haijawahi kutokea*** | ***2: Mara chache*** | ***3: Baadhi ya muda*** | ***4: Mara nyingi*** | ***5: Mara zote*** |
| **SS5. ... Kuwa na wakati mzuri na au kufanya kitu unachofurahia**  SS5. ...To have a good time with or do something you enjoy | ***1: Haijawahi kutokea*** | ***2: Mara chache*** | ***3: Baadhi ya muda*** | ***4: Mara nyingi*** | ***5: Mara zote*** |
| **SS6. ... Kwenda kwa mapendekezo juu ya jinsi ya kushughulikia tatizo**  SS6. ...To go to for suggestions on how to deal with a problem | ***1: Haijawahi kutokea*** | ***2: Mara chache*** | ***3: Baadhi ya muda*** | ***4: Mara nyingi*** | ***5: Mara zote*** |
| **SS7. ... Anayejua matatizo yako**  SS7. ...Who understands your problems | ***1: Haijawahi kutokea*** | ***2: Mara chache*** | ***3: Baadhi ya muda*** | ***4: Mara nyingi*** | ***5: Mara zote*** |
| **SS8. ... Kukupenda na kukufanya uhisi kuwa unahitajika**  SS8. ...To love you and make you feel wanted | ***1: Haijawahi kutokea*** | ***2: Mara chache*** | ***3: Baadhi ya muda*** | ***4: Mara nyingi*** | ***5: Mara zote*** |

**SS9.Je, wewe au familia yako mnahisi kama wanahitaji maelezo au mafunzo ambayo yatawasaidia kuishi nyumbani na kufanya kile mnachohitaji kufanya?**

*Do you or your family feel like they need information or training that would help you live at home and do what you need to do?*

☐No ☐ Yes→

**SS9a**. **Kama ndiyo, ni aina gani ya mafunzo au taarifa mnayohitaji?**

*If yes, what kind of training or information would you need?*

_________________________________________________________________________

_________________________________________________________________________

| **Vital Signs**  **T** ____ **RR** _____ **HR** _____ **BP** ______ / _____ **Pulse Ox** _____ **Pain** (0-100)______ **Weight** _____kg **MUAC** _____ cm  *REDCap # V1 V2 V3 V4 V5 V6 V7 V8 V9* |
| --- |

**Where is your pain?/ Maumivu yako wapi?**


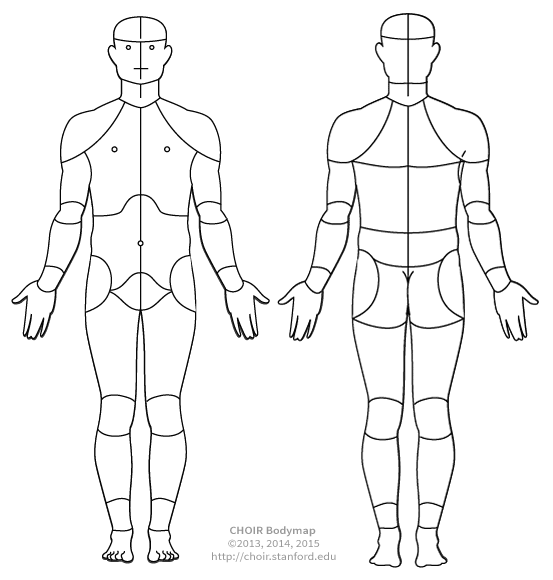


***PEG***

**1. Ni namba ipi ambayo inaelezea maumivu yako ya wastani katika wiki iliyopita?**

*What number best describes your pain on average in the past week?*

**Hakuna maumivu Mumivu mabaya ambayo hayafikiriki**

*No Pain Pain as bad as you can imagine*

| **0** | **1** | **2** | **3** | **4** | **5** | **6** | **7** | **8** | **9** | **10** |
| --- | --- | --- | --- | --- | --- | --- | --- | --- | --- | --- |

**__________________________________________________________________________________________________________________**

**2. Ni namba ipi inaelezea jinsi gani, katika wiki iliyopita, maumivu yameingilia furaha yako ya maisha?**

*What number best describes how, during the past week, pain has interfered with your enjoyment of life?*

**Hakuna maumivu Mumivu mabaya ambayo hayafikiriki**

| **0** | **1** | **2** | **3** | **4** | **5** | **6** | **7** | **8** | **9** | **10** |
| --- | --- | --- | --- | --- | --- | --- | --- | --- | --- | --- |

**__________________________________________________________________________________________________________________**

**3. Ni namba ipi inaelezea jinsi gani, katika wiki iliyopita, maumivu yameingilia shughuli zako zote?**

*What number best describes how, during the past week, pain has interfered with your general activity?*

**Hakuna maumivu Mumivu mabaya ambayo hayafikiriki**

| **0** | **1** | **2** | **3** | **4** | **5** | **6** | **7** | **8** | **9** | **10** |
| --- | --- | --- | --- | --- | --- | --- | --- | --- | --- | --- |

**__________________________________________________________________________________________________________________**

**BEHAVIORAL HISTORY; SF8**

| **SF0. Kwa kutumia mstari kama kielelezo, unawezaje kupima/kukadiria vipi hali ya afya yako kwa sasa? (Nakili kwa asilimia inayoendana na alama ya anayehojiwa)**  *From 0 to 100, how would you rate your current state of health? (Record number): __________*  *You can use the line as a guide:*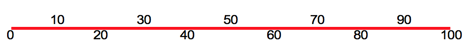 | | | | | | |
| --- | --- | --- | --- | --- | --- | --- |
| **SF1.** **Kwa ujumla, unawezaje kukadiria afya yako toka uruhusiwe kutoka hospitalini?**  *Overall, how would you rate your health* ***since discharge from the hospital****?* | *0: Bora Zaidi*  *0: Excellent* | *1: Nzuri sana*  *1: Very Good* | *2: Nzuri*  *2: Good* | *3: Inaridhisha*  *3: Fair* | *4: Duni*  *4: Poor* | *5: Hairidhishi*  *5: Very Poor* |
| **SF2.Toka uruhusiwe kutoka hospitalini, ni kiasi gani matatizo yako ya kiafya yalizuia shughuli zako za kawaida za kimwili (kama vile kutembea kwenda sokoni)?**  ***Since discharge from the hospital****, how much did physical health problems limit your usual physical activities (such as walking to the market)?* | *0: Hapana kabisa*  *0: Not at all* | *1: Kidogo sana*  *1: Very little* | *2: Kiasi*  *2: Somewhat* | *3: Sana*  *3: Quite a lot* | *4: Sikuwa naweza kufanya shughuli za kimwili*  *4: Could not do physical activities* | |
| **SF3.Toka uruhusiwe kutoka hospitalini, ulipata ugumu kwa kiasi gani katika kufanya shughuli zako za kila siku, ukiwa nyumbani au mbali na nyumbani , kwa sababu ya afya yako ya kimwili?**  ***Since discharge from the hospital****, how much difficulty did you have doing your daily work, both home and away from home, because of your physical health?* | *0: Hapana kabisa*  *0: None at all* | *1: Kidogo sana*  *1: A little bit* | *2: Kiasi*  *2: Some* | *3: Sana*  *3: Quite a lot* | *4: Sikuweza kufanya shughuli kilasiku*  *4: Could not do daily work* | |
| **SF4 Umekuwa na maumivu ya mwili kiasi gani toka uruhusiwe kutoka hospitalini?**  *How much bodily pain have you had* ***since discharge from the hospital****?* | *0: Hakuna*  *0: None* | *1: Kidogo sana*  *1: Very mild* | *2: Kidogo*  *2: Mild* | *3: Kiasi*  *3: Moderate* | *4:Makali*  *4:Severe* | *5:Makali sana*  *5: Very Severe* |
| **SF5. Toka uruhusiwe kutoka hospitalini, ulikuwa na nguvu kiasi gani?**  ***Since discharge from the hospital****, how much energy did you have?* | *0: Nyingi sana*  *0: Very much* | *1:Nyingi zaidi*  *1: Quite a lot* | *2:Kaisi*  *2: Some* | *3: Kidogo*  *3: A little* | *4:Hakuna*  *4: None* | |
| **SF6.** **Toka uruhusiwe kutoka hospitalini, ni kwa kiasi gani hali yako ya afya ya kimwili au matatizo ya kihisia yalizuia shughuli zako za kijamii na familia au marafiki?**  ***Since discharge from the hospital****, how much did your physical health or emotional problems limit your usual social activities with family or friends?* | *0: Hapana kabisa*  *0: None at all* | *1: Kidogo sana*  *1: Very little* | *2: Kidogo*  *2: Somewhat* | *3: Nyingi sana*  *3: Quite a lot* | *4: Sikuwa naweza kufanya shughuli za kijamii*  *4: Could not do social activities* | |
| **SF7.** **Toka uruhusiwe kutoka hospitalini, umesumbuliwa kwa kiasi gani na shida za mhemko (kama vile kujihisi kuwa na wasiwasi, mfadhaiko au kuwashwa)?**  ***Since discharge from the hospital****, how much have you been bothered by emotional problems (such as feeling anxious, depressed or irritable)?* | *0: Hapana kabisa*  *0: None at all* | *1: Kidogo*  *1: Slightly* | *2: Kiasi*  *2: Moderately* | *3: Nyingi sana*  *3:Quite a lot* | *4: Kuzidi kiasi*  *4: Extremely* | |
| **SF8. Toka uruhusiwe kutoka hospitalini, ni kwa kiasi gani matatizo binafsi au ya hisia yalikuzuia kufanya kazi zako za kawaida, shughuli za shule au shughuli nyingine za kila siku?**  ***Since discharge from the hospital****, how much did personal or emotional problems keep you from doing your usual work, school or other daily activities?* | *0: Hapana kabisa*  *0: None at all* | *1: Kidogo*  *1: Slightly* | *2: Kiasi*  *2: Moderately* | *3: Nyingi sana*  *3:Quite a lot* | *4: Kuzidi kiasi*  *4: Extremely* | |

**PHQ-9**:The next few questions ask about your recent mood and energy.

| **PHQ1. Toka uruhusiwe ni kwa kiasi gani umekuwa ukisumbuliwa na kutokuwa na hamu au shauku ya kufanya vitu:**  *Since discharge, how often have you been bothered by little interest or pleasure in doing things:* | *0: Hapana kabisa*  *0:Not at all* | *1:Siku kadhaa*  *1:Several days* | *2: Zaidi ya nusu ya siku zote*  *2:More than half the days* | *3: Karibu kila siku*  *3: Nearly every day* |
| --- | --- | --- | --- | --- |
| **PHQ2. Toka uruhusiwe, ni kwa kiasi gani umekuwa ukisumbuliwa na kuzubaa,kufadhaika au kukosa matumaini:**  *Since discharge, how often have you been bothered by feeling down, depressed, or hopeless:* | *0: Hapana kabisa*  *0:Not at all* | *1:Siku kadhaa*  *1:Several days* | *2: Zaidi ya nusu ya siku zote*  *2:More than half the days* | *3: Karibu kila siku*  *3: Nearly every day* |
| **PHQ3. Toka uruhusiwe, ni mara ngapi umekuwa ukisumbuliwa na matatizo ya kuanguka au kusinzia, au kulala kupita kiasi:**  *Since discharge, how often have you been bothered by trouble falling or staying asleep, or sleeping too much:* | *0: Hapana kabisa*  *0:Not at all* | *1:Siku kadhaa*  *1:Several days* | *2: Zaidi ya nusu ya siku zote*  *2:More than half the days* | *3: Karibu kila siku*  *3: Nearly every day* |
| **PHQ4. Toka uruhusiwe, ni kwa kiasi gani umesumbuliwa na kujisikia umechoka au una nguvu kidogo:**  *Since discharge, how often have you been bothered by feeling tired or having little energy:* | *0: Hapana kabisa*  *0:Not at all* | *1:Siku kadhaa*  *1:Several days* | *2: Zaidi ya nusu ya siku zote*  *2:More than half the days* | *3: Karibu kila siku*  *3: Nearly every day* |
| **PHQ5. Toka uruhusiwe, ni kwa kiasi gani umekuwa ukisumbuliwa na tatizo la kukosa hamu ya kula au kula zaidi:**  *Since discharge, how often have you been bothered by poor appetite or overeating:* | *0: Hapana kabisa*  *0:Not at all* | *1:Siku kadhaa*  *1:Several days* | *2: Zaidi ya nusu ya siku zote*  *2:More than half the days* | *3: Karibu kila siku*  *3: Nearly every day* |
| **PHQ6. Toka uruhusiwe, ni mara ngapi umekuwa ukisumbuliwa na hisia mbaya kuhusu wewe mwenyewe, au kwamba hauwezi kufanikiwa, au kuwa umejiangusha au umeiangusha familia yako**  *Since discharge, how often have you been bothered by feeling bad about yourself, or that you are a failure, or that you have let yourself or your family down:* | *0: Hapana kabisa*  *0:Not at all* | *1:Siku kadhaa*  *1:Several days* | *2: Zaidi ya nusu ya siku zote*  *2:More than half the days* | *3: Karibu kila siku*  *3: Nearly every day* |
| **PHQ7.** **Toka uruhusiwe, ni kwa kiasi gani umesumbuliwa na ugumu wa kuzingatia, kama kuzingatia unachofanya:**  ***Since discharge,*** *how often have you been bothered by trouble concentrating on things, such as what you are doing:* | *0: Hapana kabisa*  *0:Not at all* | *1:Siku kadhaa*  *1:Several days* | *2: Zaidi ya nusu ya siku zote*  *2:More than half the days* | *3: Karibu kila siku*  *3: Nearly every day* |
| **PHQ 8. Toka uruhusiwe, ni kwa kiasi gani umesumbuliwa na tatizo la kutembea au kuzungumza polepole sana**  *Since discharge, how often have you been bothered by moving or speaking so slowly that other people could have noticed. Or the opposite, being so fidgety or restless that you have been moving around a lot more than usual:* | *0: Hapana kabisa*  *0:Not at all* | *1:Siku kadhaa*  *1:Several days* | *2: Zaidi ya nusu ya siku zote*  *2:More than half the days* | *3: Karibu kila siku*  *3: Nearly every day* |
| **PHQ9. Toka uruhusiwe, ni kwa kiasi gani umekuwa ukisumbuliwa na mawazo kuwa heri kufa au kujiumiza kwa njia nyingine**  *Since discharge, how often have you been bothered by thoughts that you would be better off dead or hurting yourself in some way:* | *0: Hapana kabisa*  *0:Not at all* | *1:Siku kadhaa*  *1:Several days* | *2: Zaidi ya nusu ya siku zote*  *2:More than half the days* | *3: Karibu kila siku*  *3: Nearly every day* |

**AUDIT 3**

| **A1. Toka uruhusiwe, ni mara ngapi unatumia kinywaji kilicho na kilevi?**  *Since discharge, how often do you have a drink containing alcohol?* | *Hakuna*  *Never* | *Kila mwezi au chini ya mwezi*  *Monthly or less* | *Mara 2 hadi 4 kwa mwezi*  *2-4 times/month* | *Mara 2 hadi 3 kwa wiki*  *2-3 times / week* | *4 au zaidi kwa wiki*  *4 or more times/week* |
| --- | --- | --- | --- | --- | --- |
| **A2. Toka uruhusiwe, ni vinywaji vingapi vyenye kilevi unatumia unapokuwa unakunywa?**  ***Since discharge****, how many drinks containing alcohol do you have on a typical day when you are drinking?* | *1 au 2* | *3 au 4* | *5 au 6* | *7 au 9* | *10 au zaidi* |
| **A3. Toka uruhuiwe, ni mara ngapi unatumia vinywaji sita au zaidi kwa mara moja?**  ***Since discharge****, how often do you have six or more drinks on one occasion?* | *Haijawahi kutokea*  *Never* | *Chini ya kila mwezi*  *Less than monthly* | *Kila Mwezi*  *Monthly* | *Kwa wiki*  *Weekly* | *Kila siku au karibu kila siku*  *Daily/ almost daily* |

**Rehab/Occupational Health**

**Functional Independence Measure**

| Item | Score: | Score definitions: write 1-7 based on patient/family description of capacity |
| --- | --- | --- |
| G1. Self care | G1. | **1, Total assistance** (Subject contributes <25% of the effort or is unable to do the task)  **2, Maximal assistance** (Subject provides less than half of the effort (25-49%)  **3, Moderate assistance** (Subject still performs 50-75% of the task)  **4, Minimal assistance** (Requiring incidental hand-on help only (subject performs >75% of the task)  **5, Supervision** (Requiring only standby assistance or verbal prompting or help with set-up)  **6, Modified Independence** (Requiring the use of a device but not physical help)  **7, Complete independence** (Fully independent) |
| G6. Toileting | G6. |  |
| G10. Transfers: bed/chair/wheelchair | G10. |  |
| G14. Locomotion: walking/wheelchair | G14. |  |
| G15. Locomotion: stairs | G15 |  |
| G17. Expression | G17. |  |
| G18. Comprehension | G18. |  |
| G22. Social Interaction | G22 |  |
| G26. Problem Solving | G26 |  |
| G27. Memory | G27. |  |

**D1. Je, kuna vitu vya kimuundo nyumbani kwako kwa ajili ya kujisaidia (chooni), kuoga au vitu vingine vya matunzo ambavyo ni ngumu kwa wewe kuvifanya?**Are there **structural things in your home** that make toileting, bathing or other self-care things difficult for you to do?

☐No ☐ Yes→ **D1a .Kama ndiyo, tafadhali elezea**  If yes, please explain:

_____________________________________________________________________________________

_____________________________________________________________________________________

**D2. Je, unajihisi kuzuiwa nyumbani kwako?** Does anything stop you from leaving your home? ☐No ☐ Yes

**D3. Je, kuna kitu unatumia (Msaada / Msaidizi) ili kukusaidia unapotembea maeneo ya karibu?**

Do you use something (Assistive/Aide) to help you when you move around?

☐No ☐ Yes→ **D3a.** **Kama ndiyo, ni kipi? Chagua yote yanayohusika:** If yes, which? Check all that apply:

☐ Motorized Wheelchair/ **kiti cha magurudumu chenye mashine**

☐ Non-motorized wheelchair /**kiti cha magurudumu kisicho na mashine**

☐ Walker/ **vitembeleo** ☐ Crutches/ **Magongo ya kutembelea**

☐ Cane(s) **fimbo ya kutembelea** ☐ Prosthesis/ **Viungo bandia**

☐ Other/ **Nyingine (D3b):** _______________

**Comorbidities**

**D4. Je! Una matatizo yoyote ya matibabu?** Do you have any medical problems? ☐No ☐ Yes

**Kama ndio, Chagua yote hapo chini:** If yes, check all of the below:

D4a. Diabetes Mellitus: ☐No ☐Yes→D4a1: ☐Old diagnosis *(before injury)* ☐New diagnosis *(since discharge)*

D4b. Hypertension: ☐No ☐Yes→D4b1: ☐Old diagnosis ☐New diagnosis

D4c. Acute Kidney Injury: ☐No ☐Yes→D4c1: ☐Old diagnosis ☐New diagnosis

D4d. HIV: ☐No ☐Yes→D4d1: ☐Old diagnosis ☐New diagnosis

D4e0. Other:______________ ☐No ☐Yes→D4e1: ☐Old diagnosis ☐New diagnosis

D4f0. Other:______________ ☐No ☐Yes→D4f1: ☐Old diagnosis ☐New diagnosis

| **Follow-up Plans** | |  |  |  | **If not Attended, why not?** |
| --- | --- | --- | --- | --- | --- |
| **Co-**  **morbidity** | **Surgery Clinic**  F1 ☐No ☐Yes | **Dx** (F1a)**:** | **When** (F1b)**:**  **dd mm yyyy** | **Attended** (F1c)**:**  ☐No ☐Yes ☐Not yet | (F1d) |
|  | **Orthopedic Clinic**  F2 ☐No ☐Yes | **Dx** (F2a): | **When** (F2b)**:**  **dd mm yyyy** | **Attended** (F2c)**:**  ☐No ☐Yes ☐Not yet | (F2d) |
|  | **Medicine Clinic**  F3 ☐No ☐Yes | **Dx** (F3a): | **When** (F3b)**:**  **dd mm yyyy** | **Attended** (F3c)**:**  ☐No ☐Yes ☐Not yet | (F3d) |
|  | **Medicine Clinic #2**  F4 ☐No ☐Yes | **Dx** (F4a): | **When** (F4b)**:**  **dd mm yyyy** | **Attended** (F4c)**:**  ☐No ☐Yes ☐Not yet | (F4d) |
|  | **Medicine Clinic #3**  F5 ☐No ☐Yes | **Dx** (F5a): | **When** (F5b)**:**  **dd mm yyyy** | **Attended** (F5c)**:**  ☐No ☐Yes ☐Not yet | (F5d) |
|  | **Medicine Clinic #4**  F6 ☐No ☐Yes | **Dx** (F6a): | **When** (F6b)**:**  **dd mm yyyy** | **Attended** (F6c)**:**  ☐No ☐Yes ☐Not yet | (F6d) |
| **Substance Use** | F7 ☐No ☐Yes  F7a **______________** | **Dx** (F7b): | **When** (F7c)**:**  **dd mm yyyy** | **Attended** (F7d)**:**  ☐No ☐Yes ☐Not yet | (F7e) |
| **Mental Health** | F8 ☐No ☐Yes  F8a **______________** | **Dx** (F8b): | **When** (F8c)**:**  **dd mm yyyy** | **Attended** (F8d)**:**  ☐No ☐Yes ☐Not yet | (F8e) |
| **Rehab/OT, Pain** | **Rehab**  F9 ☐No ☐Yes | **Dx** (F9a): | **When** (F9b)**:**  **dd mm yyyy** | **Attended** (F9c)**:**  ☐No ☐Yes ☐Not yet | (F9d) |
|  | **OT**  F10 ☐No ☐Yes | **Dx** (F10a): | **When** (F10b)**:**  **dd mm yyyy** | **Attended** (F10c)**:**  ☐No ☐Yes ☐Not yet | (F10d) |
|  | **Other** (F11) ☐No☐Yes  F11a **______________** | **Dx** (F11b): | **When** (F11c)**:**  **dd mm yyyy** | **Attended** (F11d)**:**  ☐No ☐Yes ☐Not yet | (F11e) |

**Medicines**:

Current Medications: medication name, dose, frequency, **new (*since hospital discharge*) or old (*before* *injury*)**

M1 ☐__________________________________________________☐New ☐Old | ☐Take as told ☐Miss occasionally

☐Miss often

M2 ☐__________________________________________________☐New ☐Old | ☐Take as told ☐Miss occasionally

☐Miss often

M3 ☐__________________________________________________☐New ☐Old | ☐Take as told ☐Miss occasionally

☐Miss often

M4 ☐__________________________________________________☐New ☐Old | ☐Take as told ☐Miss occasionally

☐Miss often

M5 ☐__________________________________________________☐New ☐Old | ☐Take as told ☐Miss occasionally

☐Miss often

M6 ☐__________________________________________________☐New ☐Old | ☐Take as told ☐Miss occasionally

☐Miss often

**M7. Je, umekuwa na changamoto za kupata au kutumia dawa hizi?**

Have you had challenges obtaining or taking these medicines?

☐ Medicines not available / **dawa hazipatikani**

☐ Medications expensive/unaffordable **/ dawa ni ghali/kushindwa kumudu gharama**

☐They are far away **/ ziko mbali sana**

☐ Difficult to follow instructions **/ ngumu kufuata maelekezo**

☐Difficulty measuring dosage **/ ngumu kupima kipimo**

☐ Side effects (ie nausea, headache) **/ madhara (kichefuchefu,maumivu ya kichwa)**

☐ Missing needed equipment to take medicines **/ ukosefu wa vifaa vinavyohitajika kutumia dawa**

☐ ‘Tired’ of taking medicines (ie. chronic diseases) **/ kuchoka kutumia dawa (mfano magonjwa sugu)**

☐ Religious leaders/ local healers discourage medicines **/ viongozi wa dini/waganga wa jadi wanakatisha tamaa kutumia dawa**

☐ None

Patient Specific Functional Scale:

**PS0. Je, kuna shughuli zozote muhimu ambazo ungependa kuweza kuzifanya tena ambazo huwezi kufanya au ni ngumu kuzifanya kutokana na kuumia kwako?** Are there any important activities that you would like to be able to do again that you are unable to do or are having difficulty with as a result of your injury?

Score (1-10)

☐No ☐ Yes → PS1. __________________________________________________________ _______

PS2.___________________________________________________________ _______

PS3. __________________________________________________________ _______

**PS1-3a. Kama ndiyo, nioneshe kwenye kipimo hiki hali uliyonayo kwa leo kwa uwezo wako wa kufanya hivyo.** If yes, show me on this scale where you are as of today with your ability to do each activity.

**Chagua namba moja:**

| **0** | **1** | **2** | **3** | **4** | **5** | **6** | **7** | **8** | **9** | **10** |
| --- | --- | --- | --- | --- | --- | --- | --- | --- | --- | --- |

| **Hawezi kufanya shughuli kama ilivyokuwa zamani kabla ya kuumia/kupata tatizo** |  |  | **Anaweza kufanya shughuli kwa kiwango kile kile kama zamani kabla ya kuumia au kupata tatizo** |
| --- | --- | --- | --- |

**
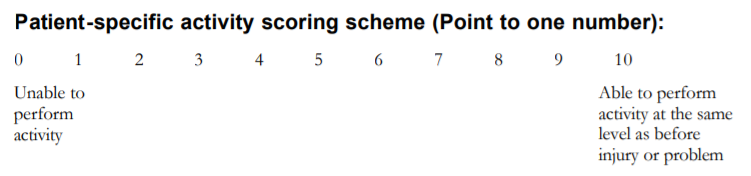
**

**CTM-15**

***Ni nani aliyefanya mahojiano?*** ☐***Mgonjwa*** ☐***Mlezi/muuguzi***

| **Melezo mafupi ya mwanzo ni kuhusu wakati uliokuwa hospitalini. . .** | | | | | |
| --- | --- | --- | --- | --- | --- |
| **CTM1. Kabla ya kuondoka hospitalini, wafanyakazi na mimi tulikubaliana juu ya malengo mazuri ya afya kwa ajili yangu na jinsi ambavyo tutayafikia malengo hayo.**  *Before I left the hospital, the staff and I agreed about clear health goals for me and how these would be reached.* | Sikubali kabisa    *Strongly Disagree* | Sikubali  *Disagree* | Nakubali  *Agree* | Nakubali kabisa    *Strongly Agree* | Sijui/Sikumbuki/Haihisiki  *Don't Know/ Remember/*  *Not Applicable* |
| **CTM2. Wafanyakazi wa hospitali walichukua mapendekezo yangu na yale ya familia yangu au mlezi kwa ajili ya kuamua ni yapi mahitaji yangu ya afya yatakayo hitajika wakati nitakapo ondoka hospitalini.**  *The hospital staff took my preferences and those of my family or caregiver into account in deciding what my health care needs would be when I left the hospital.* | Sikubali kabisa | Sikubali | Nakubali | Nakubali kabisa | Sijui/  Sikumbuki/  Haihisiki |
| **CTM3. Wafanyakazi wa hospitali walichukua mapendekezo yangu na yale ya familia yangu au mlezi kwa ajili ya kuamua ni wapi mahitaji yangu ya afya nitakapo yapata wakati nitakapoondoka hospitalini.**  *The hospital staff took my preferences and those of my family or caregiver into account in deciding where my health care needs would be met when I left the hospital.* | Sikubali kabisa | Sikubali | Nakubali | Nakubali kabisa | Sijui/  Sikumbuki/  Haihisiki |
| **Kifungu cha maelezo yanayofuata ni kuhusu wakati uipokuwa unajiandaa kuondoka hospitalini…** | | | | | |
| **CTM4. Nilipotoka hospitalini, nilikuwa na taarifa zote nilizohitaji ili kuweza kujitunza mimi mwenyewe.**  *When I left the hospital, I had all the information I needed to be able to take care of myself* | Sikubali kabisa | Sikubali | Nakubali | Nakubali kabisa | Sijui/  Sikumbuki/  Haihisiki |
| **CTM5. Nilipotoka hospitalini, nilielewa vizuri jinsi ya kusimamia afya yangu.**  *When I left the hospital, I clearly understood how to manage my health.* | Sikubali kabisa | Sikubali | Nakubali | Nakubali kabisa | Sijui/  Sikumbuki/  Haihisiki |
| **CTM6. Nilipotoka hospitalini, nilielewa wazi dalili za hatari/viashiria na dalili ninazopaswa kuziangalia kwa ajili ya kufuatilia hali yangu ya afya.**  *When I left the hospital, I clearly understood the warning signs and symptoms I should watch for to monitor my health condition.* | Sikubali kabisa | Sikubali | Nakubali | Nakubali kabisa | Sijui/  Sikumbuki/  Haihisiki |
| **CTM7. Nilipotoka hospitalini, nilikuwa na mpango ulioandikwa, unaosomeka na kueleweka kirahisi ambao unaelezea jinsi mahitaji yangu yote ya huduma ya afya yatakavyopatikana.**  *When I left the hospital, I had a readable and easily understood written plan that described how all of my health care needs were going to be met.* | Sikubali kabisa | Sikubali | Nakubali | Nakubali kabisa | Sijui/  Sikumbuki/  Haihisiki |
| **CTM8. Nilipotoka hospitalini, nilikuwa na ufahamu mzuri wa hali yangu ya afya na nini hufanya iwe bora au mbaya zaidi.**  *When I left the hospital, I had a good understanding of my health condition and what makes it better or worse.* | Sikubali kabisa | Sikubali | Nakubali | Nakubali kabisa | Sijui/  Sikumbuki/  Haihisiki |
| **CTM9. Nilipotoka hospitalini, nilikuwa na ufahamu mzuri wa mambo niliyotakiwa kuwajibika nayo katika kusimamia afya yangu.**  *When I left the hospital, I had a good understanding of the things I was responsible for in managing my health.* | Sikubali kabisa | Sikubali | Nakubali | Nakubali kabisa | Sijui/  Sikumbuki/  Haihisiki |
| **CTM10. Nilipotoka hospitalini, nilikuwa ninajiamini kwamba nilijua nini cha kufanya ili kusimamia afya yangu.**  *When I left the hospital, I was confident that I knew what to do to manage my health.* | Sikubali kabisa | Sikubali | Nakubali | Nakubali kabisa | Sijui/  Sikumbuki/  Haihisiki |
| **CTM11. Nilipotoka hospitalini, nilikuwa nnia jiamini kwamba ningeweza kufanya mambo niliyohitaji kufanya ili kutunza afya yangu.**  *When I left the hospital, I was confident I could actually do the things I needed to do to take care of my health.* | Sikubali kabisa | Sikubali | Nakubali | Nakubali kabisa | Sijui/  Sikumbuki/  Haihisiki |
| **CTM12. Nilipotoka hospitalini, nilikuwa na orodha ilioandikwa, inayosomeka na kueleweka kirahisi kwa ajili ya ahadi za kurudi hospitali au kufanya vipimo vilivyohitajika kukamilika ndani ya wiki kadhaa.**  *When I left the hospital, I had a readable and easily understood written list of the appointments or tests I needed to complete within the next several weeks.* | Sikubali kabisa | Sikubali | Nakubali | Nakubali kabisa | Sijui/  Sikumbuki/  Haihisiki |
| **Kifungu cha pili cha maelezo ni kuhusu dawa zako ...** | | | | | |
| **CTM13. Nilipotoka hospitalini, nilielewa vizuri lengo la kutumia kila dawa yangu niliyoandikiwa dawa.**  *When I left the hospital, I clearly understood the purpose for taking each of my medications.* | Sikubali kabisa | Sikubali | Nakubali | Nakubali kabisa | Sijui/  Sikumbuki/  Haihisiki |
| **CTM14. Nilipotoka hospitalini, nilielewa vizuri jinsi ya kutumia kila dawa yangu, ikiwa ni pamoja na kiasi gani napaswa kutumia na kwa wakati gani.**  *When I left the hospital, I clearly understood how to take each of my medications, including how much I should take and when.* | Sikubali kabisa | Sikubali | Nakubali | Nakubali kabisa | Sijui/  Sikumbuki/  Haihisiki |
| **CTM15. Nilipotoka hospitalini, nilielewa vizuri uwezekano wa madhara yanayoweza kusababishwa na kila dawa yangu.**  *When I left the hospital, I clearly understood the possible side effects of each of my medications.* | Sikubali kabisa | Sikubali | Nakubali | Nakubali kabisa | Sijui/  Sikumbuki/  Haihisiki |

**Z1. Je! Una maswali zaidi au maoni kwetu?** Do you have any further questions or comments for us?

**______________________________________________________________________________________________**

**______________________________________________________________________________________________**

**______________________________________________________________________________________________**

**______________________________________________________________________________________________**
