# Supplementary material for: Barriers to accessing follow up care in post-hospitalized trauma patients in Moshi, Tanzania: A mixed methods study
Source: PLOS Glob Public Health. 2022 Jun 13;2(6):e0000277. doi: 10.1371/journal.pgph.0000277 (PMC10021180; doi:10.1371/journal.pgph.0000277)
Supplement: S1 Text — (DOCX) [file pgph.0000277.s003.docx]

**COMMUNITY LEADERS FOCUS GROUP SCRIPT**

Hello, thank you for joining our session today. We want to use our time today to discuss what happens to injury patients after they leave the hospital.

**Halo, asante kwa kuhudhuria mjadala wetu wa leo. Tunataka kutumia muda wenu wa leo kujadili kinachotokea kwa wagonjwa walioumia baada ya kutoka hospitalini.**

1) How have you or your community helped support a person that was recently hospitalized because of an injury? **Je, ninyi au jamii yenu inamsaidiaje mtu aliyetoka hospitalini kwa sababu ya kuumia?**

- 1. *Probe if they are not talking:*
     1. *How could a community impact this person’s recovery?* ***Je, jamii inaweza kuathiri vipi uponyaji wa mgonjwa?***
     2. *How could a community impact/affect/help/facilitate/allow a patient’s social reintegration?* ***Je, jamii inaweza kumsaidiaje mgonjwa kurudi tena kwenye jamii na kushirikiana nao kama mwanzo?***
     3. *How could a community impact/help with a family’s adjustment?* ***Je, jamii inasaidiaje familia kurudi katika hali yake ya kawaida?***

*2) Can you describe* where (you believe) people (can) find support/help in dealing/addressing/helping with their post-injury needs? **Je, mnaweza kuelezea ni wapi watu wanaweza kupata msaada kushughulikia mahitaji yao baada ya kuumia?**

1. *Probe if they are not talking:*
   1. *What about their needs to complete/do their daily/normal activities?* ***Ni mahitaji gani wanayohitaji kwa ajili ya kufanya shughuli zao za kawaida za kila siku?***
   2. *What do you know about the community-based (in the community) support systems/resources for rehabilitation?* ***Je, mnajua nini kuhusu mifumo ya misaada ya kijamii kwa ajili ya uponyaji kwa mgonjwa?***

3) In your opinions, what are the barriers patients have/experience in getting/accessing/using rehabilitation services? **Kwa maoni yenu, wagonjwa wanapata vikwazo gani katika kupata huduma za uponyaji?**
