# Supplementary material for: Barriers to accessing follow up care in post-hospitalized trauma patients in Moshi, Tanzania: A mixed methods study
Source: PLOS Glob Public Health. 2022 Jun 13;2(6):e0000277. doi: 10.1371/journal.pgph.0000277 (PMC10021180; doi:10.1371/journal.pgph.0000277)
Supplement: S2 Text — (PDF) [file pgph.0000277.s004.pdf]

TOC MODEL FAMILY INTERVIEW SCRIPT

1) Hello, thank you for taking the time to speak with us. Today we will discuss your family member's *[ask for patient's name]* recent injury and what life has been like for all of you since *[patient's name]* returned home. **Hallo, asante kwa kutumia muda wako kuzungumza na sisi. Leo tutajadili kuhusu mwanafamilia wako** *[Uliza jina la mgonjwa]* **kuhusu kuumia kwake hivi karibuni na jinsi maisha yenu nyinyi nyote yamekuwa tangu** *[jina la mgonjwa]* **aliporudi nyumbani.**

2) I would like to start our conversation by asking you to describe how *[patient name]* injury occurred. ***Ningependa kuanza mazungumzo yetu kwa kukuomba kuelezea jinsi kuumia kwa*** *[jina la mgonjwa]* ***kulivyotokea***

   a) What was he/she doing? ***Alikuwa anafanya nini?***

      i) Was this activity related to work, leisure, or other daily activities? ***Je, shughuli hii inahusiana na kazi, burudani, au shughuli nyingine za kila siku?***

      ii) Who was he/she with? What is the *[patient's name]'s* relationship with this person? *[Brother, sister, father, mother, child, friend, spouse, colleague, other?]* ***Je alikuwa na nani? Kuna Uhusiano gani kati ya*** *[jina la mgonjwa]* ***na mtu huyo?*** *[kaka, dada, baba, mama, mtoto, rafiki, mwenzi, Mfanyakazi mwenzake, mwingine?]*

3) Would you please describe a typical day for you and [patient name] after the injury? ***Tafadhali ungependa kuelezea siku ya kawaida kwako na*** *[jina la mgonjwa]* ***baada ya kuumia?***

   a) Can you talk about [patient's name]'s needs after the injury?***Je! Unaweza kuzungumza juu ya mahitaji ya*** *[jina la mgonjwa]* ***baada ya kuumia?***

      i) If a care need is mentioned, ask: where does he/she seek care for it?*Ikiwa mahitaji ya huduma yametajwa, waulize:* ***wapi anatafuta huduma?***

      ii) If a care need is mentioned, ask: What are the difficulties in getting the care needed? *Ikiwa mahitaji ya huduma yametajwa, waulize:* ***Je! Kuna ugumu gani katika kupata huduma zinazohitajika?***

   b) *How did [patient's name]'s injury influence your family?* ***Kwa namna gani kuumia kwa*** *[jina la mgonjwa]* ***kumeathiri familia yako?***

   c) *Would you please explain how your daily activities have changed after the injury?* ***Tafadhali, unaweza kueleza jinsi shughuli zako za kila siku zimebadilika baada ya kuumia?***

   d) *How has [patient's name]'s life changed after the injury?* ***Je! Maisha ya*** *[jina la mgonjwa]* ***yamebadilikaje baada ya kuumia?***

   e) *Please describe the activities [patient's name] needs help with.*___Tafadhali elezea shughuli za___ *[jina la mgonjwa]* ***anazohitaji msaada****.*

   f) *Would you please talk more about [patient's name]'s emotional reactions after he returned home? Je, ungependa kuzungumzia zaidi kuhusu athari za kihisia za [jina la mgonjwa] baada ya kurudi nyumbani?*

   g) *How would you describe [patient's name]'s motivation for daily activities?* ***Je, unaweza kuelezeaje msukumo wa*** *[jina la mgonjwa]* ***kwa shughuli za kila siku?***

   h) *How would you describe [patient's name]'s motivation for interacting with others?* ***Je, unaweza kuelezeaje msukumo wa*** *[jina la mgonjwa]* ***wa kushirikiana na wengine?***

   i) *Would you please describe how [patient's name] has been able to self manage on his daily activities (e.g. taking a bath, moving around, changing clothers, engaging conversations)?* ***Je, ungependa kueleza jinsi*** *[jina la mgonjwa]*

ameweza kujishughulikia mwenyewe? (kama, kuoga, kutembea, kubadilisha nguo au kuvaa, kuzungumza na wengine)

j) *Would you please describe how [patient's name] has responded to simple questions that require memory, attention or complex thoughts? Have you perceived challenges?* ***Je, ungependa kuelezea jinsi*** *[jina la mgonjwa]* ***anavyojibu maswali rahisi ambayo yanahitaji kumbukumbu, kuzingatia au kufikiria sana? Umewahi kukutana na changamoto kuhusiana na hilo?***

k) Has *[patient's name]* suffered with substance use? **Je** *[jina la mgonjwa]* **huteseka na matumizi ya madawa ya kulevya au matumizi mabaya ya dawa?**

i) *If yes,* could you describe what and how is he/she using it? **Kama ndio, unaweza kuelezea ni nini na kwa namna gani yeye anaitumia?**

ii) *If yes,* when did he/she begin to use the substance(s)? **Kama ndiyo, alianza lini kutumia vitu hivyo?**

iii) *If yes,* How has his/her substance use affected your family? **Kama ndiyo, Je! Ni kwa namna gani matumizi yake yameathiri familia yako?**

l) Has *[patient's name]* reported developing any other health conditions? **Je** *[jina la mgonjwa]* **ameripoti kutokea kwa matatizo mengine ya afya?**

m) Would you like to describe any other area where your life or *[patient's name]'s* life has changed after he/she returned home? **Je! Ungependa kuelezea eneo lolote lingine kwenye maisha yako au maisha ya** *[jina la mgonjwa]* **yamebadilika baada ya kurudi nyumbani?**

4) What are the most common problems you believe that you, your family, and the affected family member are facing now that he/she had this injury? **Je! Ni matatizo gani ya kawaida unayoamini kwamba wewe, familia yako, na mwanafamilia wako aliyeumia anayapata kwa sasa kutokana na kuumia huku?**

Note to interviewer: Ask the following probing questions for each problems highlighted by the participant. If none specifically is mentioned, ask if there are any issues regarding Substance use, Pain, Mental Health, Functionality or Comorbidities (e.g. Diabetes or HIV) Zingatio kwa mhojaji: Uliza maswali yafuatayo kudadisi kwa kila matatizo yaliyoorotheshwa na mshiriki. Ikiwa hakuna liliotajwa, waulize kama kuna masuala yoyote kuhusu matumizi ya vitu, Maumivu, Afya ya akili, utendaji au magonjwa mengine (k.m. Kisukari au VVU)

a) Who supports you and your family in dealing with these difficulties *(community, family, church, work, etc)?* ***Ni nani anayekusaidia wewe na familia yako katika kukabiliana na matatizo haya*** *(jamii, familia, kanisa, kazi, nk)?*

b) How does that affect structural problems (such as transportation and accessibility)? **Je! Hiyo inaathirije usafiri na upatikanaji wa mahitaji mengine***?*

c) Would you describe the economic barriers you might be facing due to this injury? **Je! Ungependa kuelezea vikwazo vya kiuchumi ambavyo huenda unakabiliwa navyo kwasababu ya kuumia huku?**

d) How does this problem influence *[patient's name] mental health? (lack of motivation, emotional instability, isolation, loneliness) Tatizo hili linaathirije afya ya akili ya [jina la mgonjwa] (ukosefu wa motisha, ukosefu wa utulivu wa kihisia, kutengwa, upweke)*

5) Where does your family member usually go when he/she needs healthcare? **kwa kawaida, ni wapi mwanafamilia wako huenda wakati anapohitaji huduma za afya?**

a) Where does *[patient's name]* go when he/she has an emergent situation? **Je,** *[jina la mgonjwa]* **huenda wapi wakati anapokuwa na hitaji la dharura?**

b) Where does *[patient's name]* go when he/she has to seek care for chronic conditions? *(see problems reported in question #4)* **Ni wapi** *[jina la mgonjwa]* ***anakwenda kutafuta huduma kwa magonjwa ya kudumu?*** *(tazama matatizo yaliyoripotiwa katika swali la 4)*

c) Where does *[patient's name]* go when he/she needs specific healthcare assistance? **Ni wapi** *[jina la mgonjwa]* **anakwenda wakati anapohitaji msaada maalum wa afya**

d) Are you aware of any source of support in your community? **Je unajua chanzo chochote cha msaada katika jumuiya yako?**

e) Who in your community helps your family member when he/she has a problem related to the injury? **Nani katika jamii yako humsaidia mwanafamilia wako wakati ana shida kuhusiana na kuumia?**

f) What about in your family, who helps [patient's name] when he/she has a problem related to the injury? **Vipi kuhusu familia yako, ni nani anamsaidia** *[jina la mgonjwa]* **wakati ana shida inayohusiana na kuumia?**

6) Has *[patient's name]* been having activities outside of the house or with other people? **Je!** *[Jina la mgonjwa]* **amekuwa na shughuli nyingine anazozifanya akiwa na watu wengine nje ya nyumba na watu wengine?**

If YES **KAMA NDIYO:**

a) How has it been for *[patient's name]* to return to such activities after he returned home? What has he/she been able to do? With whom? **Imekuwaje kwa** *[jina la mgonjwa]* **kurudi kwenye shughuli hizo baada ya kurudi nyumbani? Je, yeye ameweza kufanya nini? Na nani?**

b) How would you describe *[patient's name]* relationships after he returned home? **Je, unaweza kuelezeaje mahusiano ya** *[jina la mgonjwa]* **baada ya kurudi nyumbani?**

c) How do you think your community perceives and addresses *[patient's name]* needs after this injury? **Je Unafikiri jumuiya yako inaonaje na kushughulikiaje mahitaji ya** *[jina la mgonjwa]* **baada ya kuumia huku?**

d) How has your family member been accepted or received back in your community after the injury, given the new changes in his/hers life? **Je ni kwa namna gani mwanafamilia wako amekubaliwa au kupokelewa tena katika jamii yako baada ya kuumia, kutokana na mabadiliko mapya katika maisha yake?**

e) What are the problems you have noticed with *[patient's name]* after he started to get involved in activities out of the house? **Je, ni matatizo gani uliyoona kwa** *[jina la mgonjwa]* **baada ya kuanza kushiriki katika shughuli za nje ya nyumba?**

If NO **KAMA HAPANA:**

f) How would you describe *[patient's name]* barriers to being involved in activities after he/she returned home? **Je, unaweza kuelezeaje vikwazo vya** *[jina la mgonjwa]* **kushiriki katika shughuli baada ya kurudi nyumbani?**

g) What are the problems you have noticed with *[patient's name]* after he/she started to get involved in activities out of the house? **Je! Ni matatizo gani uliyoyaona kwa** *[jina la mgonjwa]* **baada ya kuanza kufanya kazi za nje ya nyumba?**

7) Thank you for your participation. To finish this interview, is there anything else you would like to say about the questions I asked you? **Asante kwa ushiriki wako. Ili kumaliza mahojiano haya, kuna kitu kingine chochote ungependa kusema kuhusu maswali niliyokuuliza?**
